# Iboga Inspired *N*-Indolylethyl-Substituted Isoquinuclidines as a Bioactive Scaffold: Chemoenzymatic Synthesis and Characterization as GDNF Releasers and Antitrypanosoma Agents

**DOI:** 10.3390/molecules27030829

**Published:** 2022-01-27

**Authors:** Mariana Pazos, Estefania Dibello, Juan Manuel Mesa, Dalibor Sames, Marcelo Alberto Comini, Gustavo Seoane, Ignacio Carrera

**Affiliations:** 1 Laboratorio de Síntesis Orgánica, Departamento de Química Orgánica, Facultad de Química, Universidad de la República, General Flores 2124, Montevideo 11800, Uruguay; mpazos@fq.edu.uy (M.P.); edibello@fq.edu.uy (E.D.); jmmesa@fq.edu.uy (J.M.M.); gseoane@fq.edu.uy (G.S.); 2 Group Redox Biology of Trypanosomes, Institut Pasteur de Montevideo, Mataojo 2020, Montevideo 11400, Uruguay; mcomini@pasteur.edu.uy; 3 Department of Chemistry, Columbia University, New York, NY 10027, USA; ds584@columbia.edu

**Keywords:** iboga alkaloid, isoquinuclidine, toluenedioxigenase, imino Diels–Alder, GDNF, anti-trypanosoma

## Abstract

The first stage of the drug discovery process involves the identification of small compounds with biological activity. Iboga alkaloids are monoterpene indole alkaloids (MIAs) containing a fused isoquinuclidine-tetrahydroazepine ring. Both the natural products and the iboga-inspired synthetic analogs have shown a wide variety of biological activities. Herein, we describe the chemoenzymatic preparation of a small library of novel *N*-indolylethyl-substituted isoquinuclidines as iboga-inspired compounds, using toluene as a starting material and an imine Diels–Alder reaction as the key step in the synthesis. The new iboga series was investigated for its potential to promote the release of glial cell line-derived neurotrophic factor (GDNF) by C6 glioma cells, and to inhibit the growth of infective trypanosomes. GDNF is a neurotrophic factor widely recognized by its crucial role in development, survival, maintenance, and protection of dopaminergic neuronal circuitries affected in several neurological and psychiatric pathologies. Four compounds of the series showed promising activity as GDNF releasers, and a leading structure (compound **11**) was identified for further studies. The same four compounds impaired the growth of bloodstream *Trypanosoma brucei brucei* (EC_50_ 1–8 μM) and two of them (compounds **6** and **14**) showed a good selectivity index.

## 1. Introduction

**Figure 1:**
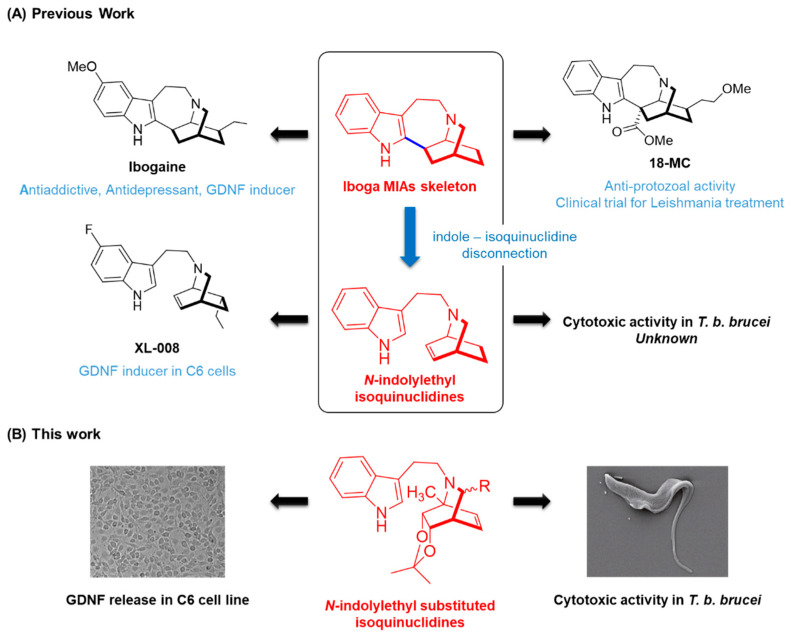
Iboga alkaloids have been investigated for their variety of pharmacological effects, such as induction of GDNF expression in the central nervous system (ibogaine) and anti-protozoal activity (18-MC). In this work we study these activities in inspired simplified analogs such as enantiomerically pure *N*-indolylethyl-substituted isoquinuclidines.

Indole alkaloids have been a large source of bioactive compounds for drug discovery. Specifically, iboga alkaloids are a group of monoterpene indole alkaloids (MIAs) containing a fused isoquinuclidine-tetrahydroazepine ring, that have been investigated for their wide variety of fascinating pharmacological effects. Ibogaine, the main representative of the group, is a potent atypical psychedelic agent with promising potential to treat substance use disorders (SUD), as evidenced by preclinical self-administration animal models and open-label studies in humans (Figure 1) [1]. We recently reported that ibogaine induces a dose-dependent upregulation of glial cell-derived neurotrophic factor (GDNF) in specific rat brain regions related to the mesocorticolimbic and nigral dopaminergic circuits [2], an effect which was previously proposed as a molecular mechanism underlying the attenuation of drug self-administration and drug craving by ibogaine [3]. GDNF is a neurotrophic factor crucial for the development, survival, maintenance, and protection of dopaminergic, noradrenergic, cholinergic, and motor neurons [4,5]. Moreover, alteration in GDNF expression has been related to several neurological pathologies, such as SUD, Parkinson’s disease, epilepsy, and neuropathic pain, among others [6,7,8]. Therefore, GDNF qualifies as a promising target to treat neuropathologies [4,9,10]. In this context, the development of small molecules, brain-permeable and capable of inducing GDNF expression in the CNS, has attracted interest [11,12,13]. Previous reports in the literature showed that *N*-indolylethyl-substituted isoquinuclidines with disconnected heteroarene and isoquinuclidine systems of the iboga skeleton act as potent GDNF-releasers in the C6 glioma cell line [14,15]. This cell line is commonly used as an astrocyte model [16,17] that is able to express GDNF in response to different stimuli, including a variety of small molecules [13,18,19,20,21,22,23]. From these series, compound XL-008 (Figure 1) was reported as the most potent GDNF releaser in C6 cells [15].

Additionally, several iboga alkaloids such as coronaridine [24], heyneanine [25], voacangine [24], the synthetic analog 18-MC [26], and iboga *bis*-indole alkaloids (voacamine [27,28], conodurine, gabunine, and conoduramine [29]) have shown promising in vitro activities as anti-protozoal agents to treat trypanosomiasis (i.e., African trypanosomiasis and Chagas disease) and leishmaniasis (cutaneous, mucocutaneous, or visceral forms) (Figure 1). Worth noting is that a phase II clinical trial to evaluate the tolerability, safety, and efficacy of 18-MC as drug candidate for tegumentary leishmaniasis is ongoing [30]. To our knowledge, there are no previous studies reporting the potential anti-protozoal activity of *N*-indolylethyl-substituted isoquinuclidines as simplified iboga analogs. 

In this manuscript, we envisioned the chemoenzymatic preparation of novel enantiopure highly functionalized *N*-indolylethyl-substituted isoquinuclidines as iboga-inspired compounds. Toward this aim, we used toluene as a starting material to produce an enantiopure *cis*-cyclohexadienediol **1** using the toluene dioxygenase enzymatic complex (TDO). Compound **1** acted as the diene in an aza Diels–Alder reaction to construct the isoquinuclidine system, which was further coupled to the *N*-indolylethyl residue. The ability of these novel derivatives to promote the release of GDNF by C6 glioma cells, and to inhibit the proliferation of infective African trypanosomes, was studied. 

## 2. Results

### 2.1. Chemoenzymatic Synthesis of Highly Functionalized N-Indolylethyl Isoquinuclidines

**Figure 2:**
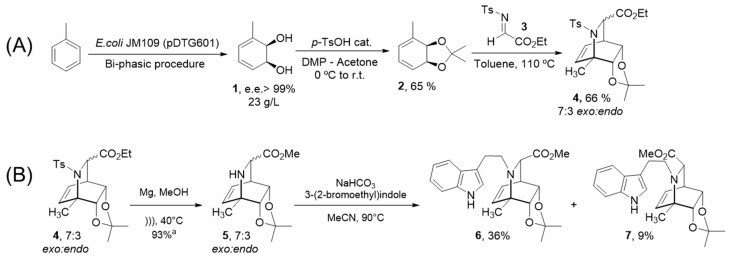
(**A**) Chemoenzymatic synthesis of the isoquinuclidine core **4** using toluene as starting material. (**B**) Preparation of *N*-indolylethyl isoquinuclidines **6** and **7**. ^a^ Yield estimated by ^1^H-NMR of the crude reaction using trichloroethylene as internal standard.

Our first objective was to prepare a small library of novel highly functionalized *N*-indolylethyl-substituted isoquinuclidines. A stereoselective two-step protocol previously developed by our group was applied for the synthesis of isoquinuclidines [31]. This synthetic strategy uses toluene as starting material, which is enzymatically transformed into the corresponding *cis*-cyclohexadienediol with high yields as a single enantiomer (Figure 2). This biotransformation, carried out using the toluene dioxygenase enzymatic complex expressed in recombinant form by *E. coli* JM109(pDTG601), has been carefully optimized by our laboratory using a bi-phasic protocol in a 5 L bioreactor, yielding 23 g of dienediol per liter of culture broth [32]. Protection of the diol system in **1** with the isopropylidene group using standard conditions to obtain **2**, followed by an aza Diels–Alder reaction with the tosylimine derived from ethyl glioxylate **3** as the dienophile, rendered the isoquinuclidine core of interest **4** with 66% yield as an inseparable diasteromeric mixture of products exo:endo 7:3 (Figure 2A) [31].

In order to couple the indolyl ethyl residue at the nitrogen atom of the isoquinuclidine framework, the tosyl group needed to be removed. Several conditions for sulfonamide removal were assayed (see Supplementary Materials Table S1); the best results were those using Mg and ultrasound in methanol, which rendered the free amines **5** as their methyl esters (because of transesterification with the solvent). These amines turned out to be highly unstable and extensive decomposition was detected during their purification using standard chromatographic methods. Because of this, the next coupling reaction with the 3-(2-bromoethyl)indole group was carried out using the crude reaction without further purification. From the different bases and conditions assayed for the coupling reaction (see Supplementary Materials Table S2), NaHCO_3_ in MeCN at 90 °C in a sealed tube gave the best results, affording products **6** and **7** in 45% overall yield (8:2 ratio), which were separable using silica chromatography (Figure 2B). 

**Figure 3:**
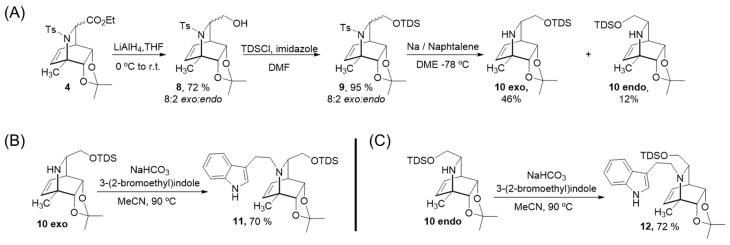
(**A**) Preparation of free amines **10 exo** and **10 endo** from esters mixture **4.** (**B**) Preparation of derivative **11**. (**C**) Preparation of derivative **12**.

In order to diversify the structures of the series, the esters in the mixture **4** were reduced using LAH to render the corresponding alcohols **8** in 72%, which were protected using chloro(dimethyl)thexylsilane (TDSCl) to afford the corresponding silyl ethers in 95% yield. The use of this large and non-polar protective group allowed the separation of the *exo* and *endo* diasteromers in the following steps in order to get pure stereoisomers for biological evaluation (Figure 3A). Tosyl deprotection of compounds 9 using conditions involving sodium naphtalide (which in this case were superior to the magnesium ultrasound-assisted protocol used before) rendered the free amines 10 in an 8:2 *exo: endo* ratio (58% overall yield) that were easily separated using flash silica chromatography. Coupling with the 3-(2-bromoethyl)indole using previously optimized conditions produced the derivatives **11** and **12** in 70% and 72% yield, respectively (Figure 3B,C).

**Figure 4:**
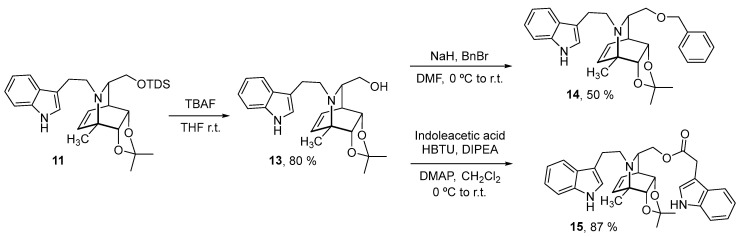
Synthesis of compounds **14** and **15** using analog **11** as starting material.

Next, compound **11** was used as a starting material to synthetize new analogs. As shown in Figure 4, deprotection of the silyl group using TBAF in THF gave alcohol **13** in 80% yield. From this alcohol, the benzylether **14** and the indol-ester **15** were synthetized in 50% and 87% yield, respectively, using standard conditions for benzylation and HBTU as a coupling agent for the ester formation.

In this manner, six enantiomerically-pure highly functionalized *N*-indolylethyl isoquinuclidines were synthesized from toluene in five to six reaction steps, with global yields ranging from 5% to 20%. Next, biological evaluation as potential GDNF releasers and anti-protozoal drugs was carried out.

### 2.2. Evaluation of the N-Indolylethyl Isoquinuclidines as GDNF Inducers

**Figure 5:**
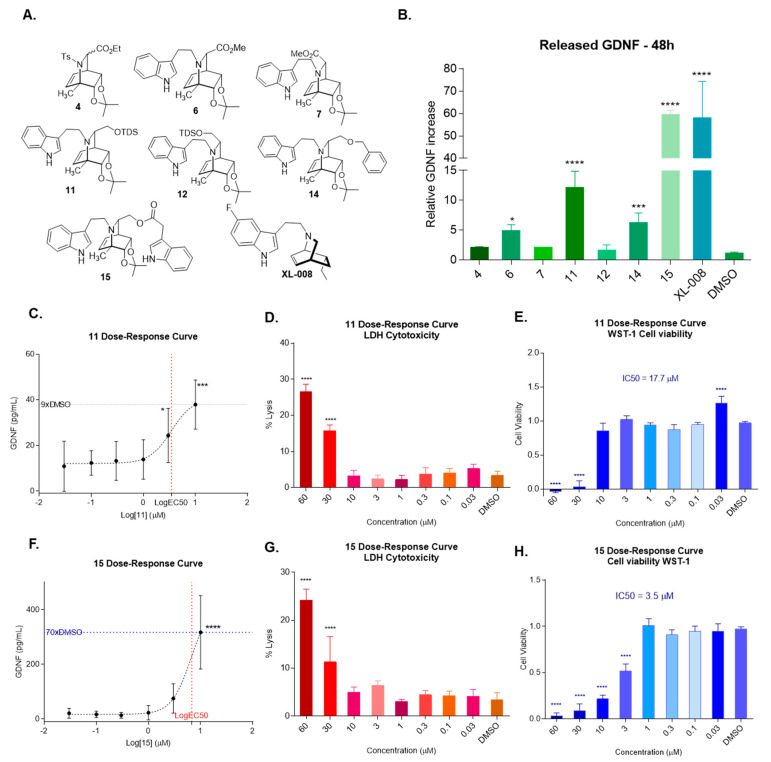
(**A**) Chemical structure of iboga-like compounds tested on their ability to promote GDNF release. (**B**) GDNF released by C6 cells after 48 h treatment with compounds shown in Figure 5A at 10 µM concentration. (**C**) **11** dose-response curve after 48 h treatment. (**D**) **11′**s effect on cell lysis determined by LDH assay. (**E**) **11′**s effect on cell viability determined by WST-1 assay. (**F**) **15′**s dose-response curve after 48 h treatment. (**G**) **15′**s effect on cell lysis determined by LDH assay. (**H**) **15′**s effect on cell viability determined by WST-1 assay. **Statistics:** data represent mean ± SEM of 3 biological replicates. One-way ANOVA followed by Dunnett’s multiple comparisons test is shown (* *p* < 0.05, *** *p* < 0.001, **** *p* < 0.0001).

An initial screening was carried out using confluent C6 cells treated with compounds **4**, **6**, **7**, **11**, **12**, **14**, and **15** (Figure 5A) at 10 μM concentration for 48 h (no appreciable GDNF release was detected for 24 h for the studied compounds, data not shown) using **XL-008** (10 μM) and DMSO as positive and negative controls, respectively (Figure 5B). After incubation, the GDNF released to culture media was quantified using a standard sandwich ELISA assay. As shown in Figure 5B, compound **15** increased the GDNF in the medium to a level up to 60 times higher than in the negative control, this activity being comparable to that of **XL-008** (GDNF/DMSO ~ 57 according to our experiment). Compounds **6**, **11**, and **14** also induced a significant release of GDNF (up to 6, 15, and 7 times higher than in the negative control, respectively).

For these four compounds (**6**, **11**, **14**, and **15**), dose-response assays (48 h exposure at concentrations from 0.03 to 60 µM) were performed to determine effective and non-toxic concentrations (Figure 5C–H). The lactate dehydrogenase (LDH) and the WST-1 assays were used to assess cell death (lysis) and cell viability, respectively. 

Compound **11** showed a good dose-response correlation (Figure 5C), with maximum response at 10 µM, producing a GDNF release 9 times higher than the vehicle. Above this concentration, a marked decrease in GDNF release was detected, which can be attributed to the significant cell death (Figure 5D) and impairment in cell viability (Figure 5E) observed at 30 and 60 µM. The half-maximal effective concentration (EC50) corresponding to the release of GDNF was calculated as 3.46 µM whereas the half-maximal inhibitory concentration (IC50) of cell proliferation was estimated as 17.7 µM. Thus, compound **11** showed a good range of effective concentrations at which it did not exert cytotoxic effects, emerging as a possible leader (see Discussion section).

Compound **15** showed a good dose-response profile, with an EC50 estimated in 6.79 µM and maximum GDNF release at 10 µM that was 70 times higher than that of the vehicle (Figure 5F). However, and similar to **11**, a marked decrease in GDNF release was observed above this concentration, which correlated with a significant percentage of cell cytotoxicity (IC50 = 3.5 µM; Figure 5G, H). Thus, the potent GDNF-inducing activity of **15** overlaps it high cytotoxicity. 

In the dose-response study for compound **6**, a significant increase in GDNF was observed only at two specific concentrations (Figure S2A): 30 and 0.03 µM, which was surprising since the initial screening showed statistically significant GDNF increase at 10 µM concentration. Since cell viability is affected for all concentrations above or equal to 10 µM (Figure S2B), the GNDF increase detected at 10 µM (Figure 5B) and 30 µM (Figure S2A) could be a consequence of a toxic response. Additionally, cell lysis was detected only at the highest concentration, 60 µM (Figure S2C). In contrast, the significant increase in GDNF release at 0.03 µM seems to be specific and unrelated to cytotoxicity (Figure S2B). Future studies with compound **6** will aim at performing dose-response curves spanning a lower range of concentrations.

Finally, regarding compound **14** we observed a maximum effect at 10 µM, a value 23 times higher than that of the vehicle (Figure S3A). At higher concentrations of **14**, GDNF was not detected, which can be ascribed to an impairment in cell viability (Figure S3B, C) at concentrations as low as 300 nM. Cell lysis was significant at 30 and 60 µM. The EC50 for 14 was estimated to be 20.11 µM, hence within the cytotoxicity range. These results ruled out this compound as a possible head of series for GDNF release since active concentrations overlap with cytotoxic effects.

### 2.3. Evaluation of the N-Indolylethyl Isoquinuclidines as Potential Anti-Trypanosomal Agents

As part of our search for candidate compounds to develop novel antiparasitic drugs, the biological activity against bloodstream *T. brucei brucei* was investigated for compounds **6**, **7**, **10 exo**, **11**, **13**, **14**, and **15** [33]. In order to estimate the biological selectivity, the cytotoxicity towards murine macrophages (cell line J774) was tested for the most active compounds (i.e., EC50 vs. *T. brucei* <10 μM). In order to compare the activity of the tested compounds with previously synthetized *N*-indolylethyl-substituted isoquinuclidines analog, the EC50 of **XL-008** was determined. 

A preliminary screening performed at 10 µM revealed five compounds reducing parasite viability to <50%, whereas **10 exo** (cell viability 64%) and **13** (cell viability 88%) were less active. The most active hits exhibited the following order of potency (EC50): **14** (1.3 μM) > **15** (3.5 μM) > **6** = **11** (7.9 μM) > **7** (31.5 μM), which is within the same order of magnitude to that displayed by the clinical drug Nifurtimox (EC50 5.3 μM; see Table 1 and Supplementary Material for dose-response curves). Notably, four of the novel *N*-indolylethyl isoquinuclidines (**6**, **11**, **14** and **15**) were 2.5- to 15-fold more potent than the related compound **XL-008** (EC50 = 20 µM).

The selectivity index (SI) of the compounds displayed the following order: **14** (30) > **6** (>12) > **15** (6) > **11** (4). Thus, the most potent iboga-like compound from the new series was the most selective one. 

## 3. Discussion

The results obtained regarding GDNF allowed us to identify analogs **6**, **11**, **14**, and **15** as inducers of GDNF-release in C6 cells exposed for 48 h to the compounds at 10 µM. Additionally, the study of their dose-response profiles and the corresponding toxicity gave rise to the identification of **11** as a promising leading structure with non-overlapping EC50 and IC50. This was not the case for **14** and **15**, which were toxic at their GDNF-active concentrations. Compound **6** also was an interesting candidate that will be subjected to further studies aiming to determine its potential to induce GDNF-release at low to sub-nM concentrations.

Our results show the importance of discriminating stress response (at cytotoxic concentrations) from specific signaling for GDNF-release. This is deemed crucial when searching for bioactive compounds of this pathway because release of neurotrophic factors such as GDNF is upregulated upon injury and in response to neurotoxic conditions in the CNS [13,34]. In line with this, the most active compound, 15, was shown to be the most cytotoxic one. In our study, structure 11 appears as a promising leader that stimulates GDNF release at concentrations that are not cytotoxic for C6 cells. This differs from the behavior reported in the literature for XL-008 in similar experimental conditions.

Despite the small number of iboga analogs tested, it is possible to identify some molecular determinants of activity that may guide the design of novel active and non-toxic GDNF releasers. For instance, the differences in the biological activity of stereoisomers **6** vs. **7** and **11** vs. **12** highlight the importance of the stereochemistry in that specific position (see Figure 5A). Moreover, compounds with a protected alcohol (**11**, **14**, and **15**) rather than an ester function (**6** and **7**) performed better as GDNF releasers, and among such protective groups, the silylated derivative showed the best activity profile. Taking these results into account, additional protective groups will be tested in the future as well as free alcohol 13 to establish further SARs. Finally, it is important to highlight that among the most bioactive compounds, the bis-indolic derivative (**15**) proved to be more cytotoxic towards mammalian cells (macrophages and glioma cells) than the mono-indolic one (**14**). In this respect, we hypothesize that the release of the indole acetate moiety intracellularly and its subsequent oxidation could be responsible for its cytotoxicity [35]. An interesting and discrete modification of 15 that may turn it into a non-hydrolysable bis-indolic derivative with lower cytotoxicity but yet a GDNF-inducer would be to change the ester for an ether function.

Regarding the anti-trypanosomal activity of the iboga derivatives, the most GDNF-active compounds were shown to be the most potent in killing the infective stage of *T. brucei*. Interestingly, the substitution with the ethylindole moiety at the nitrogen of compound **10exo** generated a derivative, **11** (EC50 of 6.95 µM), with improved activity. Regarding stereochemistry aspects, *exo* compound **6** showed a better activity than *endo* compound **7**. Compounds **11**, **14**, and **15**, where the hydroxyl group is protected with different groups, were active. Comparing compounds **14** and **15** with compounds **6** and **8**, it seems that an additional aromatic ring in the structure increases potency but also cytotoxicity against mammalian cells. Worth noting is that several of the compounds synthesized by us (**6**, **11**, **14** and **15**) proved much more active than a previous one (**XL-008**). From the compounds tested, the iboga analog **14** emerges as an interesting candidate for developing a new series of potential anti-trypansomal compounds, and future studies will address modifications of its structure in order to improve its activity. 

## 4. Materials and Methods

### 4.1. Chemoenzymatic Synthesis of Iboga-Inspired Compounds

*General considerations*

Chemicals and reagents were purchased from Sigma-Aldrich and used as received. All solvents were distilled prior to use. NMR spectra were obtained in CDCl_3_ on a Bruker Avance DPX-400 instrument. Proton chemical shifts (δ) are reported in ppm downfield from TMS as an internal reference, and carbon chemical shifts are reported in ppm relative to the center line of the CDCl_3_ triplet (77.0 ppm). Optical rotations were measured on a Zuzi 412 polarimeter using a 0.5 dm cell and on a Dichrom P-2000 polarimeter using a 3.5 mm × 100 mm cell. [α]_D_ values are given in units of deg·cm^2^·g^−1^ and concentration values are expressed in g/100 mL. High-resolution mass spectra were obtained on a Bruker Daltonics Q-TOF spectrometer (ESI mode) and on a Thermo Scientific Q Exactive Plus. Infrared spectra (IR) were recorded either on neat samples (KBr or NaCl disks) or in solution on a Shimadzu FT-IR 8101A spectrophotometer. Analytical TLC was performed on silica gel 60F-254 plates and visualized with UV light (254 nm) and/or p-anisaldehyde in acidic ethanolic solution. Flash column chromatography was performed using silica gel (Kieselgel 60, EM reagent, 230–400 mesh).

*Synthetic procedures and spectroscopic data*

*Biotransformation using E. coli JM109 (pDTG601) to produce dienediol **1***. Growth and biotransformation in the bioreactor using *E. coli* JM109 (pDTG601) were carried according to our previously published procedure [31,32]. Briefly, 5mL of LB medium supplemented with ampicillin sodium salt (0.1 g/L) and glucose (5 g/L) was inoculated with a single colony of *E. coli* JM109 (pDTG601), and grown overnight at 37 °C and 150 rpm. Two 500 mL shake-flasks containing 150 mL of MSB medium were inoculated with 1 mL of the grown culture. These preculture flasks were placed in an orbital shaker at 37 °C and 150 rpm, for 12 hrs. Both entire cultures were used to inoculate the bioreactor (Sartorius Biostat A plus), charged with an initial volume of 2.5 L, and set to 500 rpm, 30 °C, and air flow rate of 4 L/min. The pH value was controlled automatically to 6.8 by addition of conc. ammonium hydroxide during the whole process. A pulse of antifoam agents (Aldrich’s Antifoam Y: Silicone dispersion in water 1:1) was added at the beginning of the run. At 6 h after inoculation the dissolved oxygen value sharply increased (indicating carbon deprivation), whereupon a glucose-fed batch was started by adding glucose (0.7 g/mL solution) from an initial rate of 0.08 mL/min to 0.54 mL/min in a 20 h period. When the biomass concentration reached 15 g/L cdw, IPTG was added to induce TDO expression (IPTG final concentration in bioreactor of 10 mg/L), and the stirrer speed was set to 900 rpm. After the culture reached the stationary phase (c.a. 26 h, 50 g/L cdw approx.), glucose feeding was decreased to 0.25 mL/min and substrate addition was started. A solution of toluene in liquid paraffin (0.5 M) was added at a flow rate of 20 mL/min using a peristaltic pump. After the biotransformation was completed, the pH of the medium in the bioreactor was adjusted to 7.5. The culture broth was centrifuged at 7000 rpm and 4 °C for 30 min, the supernatant was collected, and the cell pellet properly disposed of. Centrifugation allows the separation of the liquid paraffin (which contains no detectable amounts of products) from the aqueous phase. The latter was properly lyophilized overnight to obtain a dry powder which was extracted several times with ethyl acetate until no more diol was detected by TLC. Solvent was evaporated to afford the corresponding dienediol 1 which was washed several times with hexanes to remove liquid paraffin traces.

**(1S**,**4S**,**7R**,**8S)-Ethyl 7**,**8-isopropylidendioxy-1-methyl-2-tosyl-2-azabicycle[2 .2.2]5-octenen-3-carboxylate (4**, **7:3 *exo:endo*):**

Ethyl glyoxylate was dissolved in dry toluene, followed by *p*-toluenesulphonyl isocyanate (1 equiv.) and catalytic amounts of AlCl_3_ (0.5% m/m of p-toluenesulphonylisocyanate) (final *p*-toluenesulphonylisocyanate (3) concentration = 2 M). The mixture was heated at reflux for 4.5 h, after which it was cooled to 50 °C and used directly in the cycloaddition reaction. A 0.5 M solution of diene 2 in dry toluene was added to the freshly prepared *N*-tosyl imine. The mixture was heated at reflux until the starting material was consumed. The system was allowed to reach room temperature. The solvent was evaporated under reduced pressure and the crude was purified by SiO_2_ column chromatography using Hex(9):AcOEt(1) as mobile phase, R = 66%.

**4 exo: ^1^H-NMR** (400 MHz, CDCl_3_): δ (ppm) = 7.96 (dt, *J* = 1.9, 8.3 Hz, 2H), 7.30 (d, *J* = 8.2 Hz, 2H); 6.22 ddd, *J* = 0.9, 6.7, 8.0 Hz, 1H), 5.96 (dt, *J* = 1.1, 8.1 Hz, 1H), 4.36 (ddd, *J* = 0.7, 3.5, 7.1 Hz, 1H), 4.31 (q, *J* = 7.2 Hz, 2H), 4.24 (d, *J* = 3.3 Hz, 1H), 4.16 (dd, *J* = 0.9, 7.1 Hz, 1H), 3.40 (dddd, *J* = 1.2, 3.4, 3.4, 6.7 Hz, 1H), 2.42 (s, 3H), 1.44 (s, 3H), 1.35 (t, *J* = 7.2 Hz, 3H), 1.25 (s, 3H), 1.23 (s, 3H); **^13^C-NMR** (100 MHz, CDCl_3_): δ (ppm) = 170.7, 135.7, 129.5, 128.0, 110.0, 81.0, 73.6, 61.7, 58.9, 58.7, 37.6, 25.5, 25.3, 21.6, 19.2, 14.2; **4 endo: ^1^H-NMR** (400 MHz, CDCl_3_): δ (ppm) = 8.09 (dt, *J* = 1.9, 8.3 Hz, 2H), 7.30—7.28 (m, 2H); 6.07 (ddd, *J* = 0.9, 6.4, 7.6 Hz, 1H), 5.91 (ddd, *J* = 1.0, 1.6, 8.0 Hz, 1H), 4.60 (d, *J* = 2.4 Hz, 1H), 4.58 (dd, *J* = 1.0, 7.1 Hz, 1H), 4.53 (ddd, *J* = 1.0, 3.3, 7.1 Hz, 1H), 4.25—4.23 (dd, *J* = 0.9, 7.1 Hz, 1H), 3.52—4.48 (m, 1H), 2.42 (s, 3H), 1.39 (s, 3H) 1.32—1.27 (m, 9H); **^13^C-NMR** (100 MHz, CDCl_3_): δ (ppm)= 170.72, 143.49, 140.28, 136.43, 129.56, 129.46, 128.13, 109.64, 79.07, 76.04, 61.48, 58.78, 56.27, 39.75, 29.69, 26.90, 25.48, 22.66, 19.23; **IR** (4 exo + 4 endo) ν_max._ (cm^−1^): 2986, 2937, 1746, 1375, 1118, 712; **MS** (4 exo + 4 endo) (EI, 70eV) *m*/*z* (%) = 65.1 (22); 91.1 (98); 155.1 (58); 248.1 (100); 406.1 (6); **HRMS** (ESI+) calc. for C_21_H_27_NO_6_Sna = 444.1457 (M+Na+) found 444.1451.

**(1*S***, **4*S***, **5*R***, **6*S*)-Methyl 1-methyl-5**,**6-isopropylidendioxy-2-azabicycle[2.2.2]-7-octenen-3-carboxylate (5**, **7:3 *exo:endo*):**

A solution of 4 in MeOH (12 mL/mmol of 4) and Mg shavings (5 equiv. previously activated with HCl ac. 5%) was stirred under ultrasound at 40 °C for three hours, or total consumption of the starting material. Once the reaction was completed, it was diluted with CH_2_Cl_2_ and washed with a 0.5 M aqueous solution of HCl. The organic phase was washed two times with 1 M aqueous solution of NaHCO_3,_ and with brine. Then, it was dried over Na_2_SO_4_ and the solvent was evaporated under reduced pressure. Yield was determined by ^1^H-NMR using trichloroethylene as internal standard. Instability of the product made its isolation and purification difficult. So, the full characterization was made over its derivative 6, yield = 93%.

**5 endo: ^1^H-NMR** (400 MHz, CDCl_3_): δ (ppm) = 6.09 (dt, *J* = 8.2, 1.3 Hz, 1H), 5.99—5.92 (m, 1H), 4.39 (ddd, *J* = 7.2, 3.4, 1.2 Hz, 1H), 4.02 (dd, *J* = 7.1, 1.2 Hz, 1H), 3.73 (s, 3H), 3.52 (d, *J* = 2.2 Hz, 1H), 3.34—3.30 (m, endo, 0.3H), 1.42 (s, 3H), 1.32 (s, 3H), 1.29 (s, 3H). **5 exo: ^1^H-NMR** (400 MHz, CDCl_3_): δ (ppm)= 6.24 (ddd, *J* = 7.8, 6.5, 1.1 Hz, 1H), 6.05 (dt, *J* = 8.2, 1.2 Hz, 1H), 4.19 (ddd, *J* = 7.3, 3.5, 1.1 Hz, 1H), 3.98 (dd, *J* = 7.2, 1.1 Hz, 1H), 3.79 (s, 3H), 3.40 (d, *J* = 2.6 Hz, 1H), 3.28 (dddd, *J* = 6.2, 3.7, 2.6, 1.3 Hz, 1H), 1.42 (s, 3H), 1.31 (s, 3H), 1.26 (s, 3H).

**(1*S***, **3*R***, **4*S***, **5*R***, **6*S*)-Methyl *N*-(2-(3-indolyl)ethyl)-1-methyl-5**,**6-isopropylidendioxy-2-azabicycle[2.2.2]-7-octenen-3-carboxylate(6) and(1*S***, **3*S***, **4*S***, **5*S***, **6*R*)-Methyl *N*-(2-(3-indolyl)ethyl)-1-methyl-5**,**6-isopropylidendioxy-2-azabicycle[2.2.2]-7-octenen-3-carboxylate(7)**

A 0.25 M solution of **5** in acetonitrile with 3-(2-bromoethyl)indole (1.1 equiv.) and NaHCO_3_ (4 equiv.) was heated to 90 °C in a sealed tube until total consumption of the starting material. Once the reaction was completed, the crude was diluted with water and extracted three times with CH_2_Cl_2._ The organic phase was washed with a 10% aqueous solution of NaHCO_3_ and dried over Na_2_SO_4_. The solvent was evaporated under reduced pressure and the crude (*exo*(8):*endo*(2) mixture) was purified by SiO_2_ column chromatography using Hex(7):AcOEt(3) as mobile phase. Compound **6** was obtained with a 36% yield and compound **7** with a 9% yield. Given the high instability of **5**, compound **7** was obtained only once and in small quantities, hence full characterization was not possible.

**6: ^1^H-NMR** (400 MHz, CDCl_3_): δ (ppm) = 8.01 (s, 1H), 7.57 (ddd, *J* = 8.0, 2.0, 0.9 Hz, 1H), 7.37 (dt, *J* = 8.0, 1.0 Hz, 1H), 7.20 (ddd, *J* = 8.2, 7.0, 1.2 Hz, 1H), 7.13 (ddd, *J* = 8.0, 7.0, 1.2 Hz, 1H), 7.00 (d, *J* = 2.4 Hz, 1H), 6.29 (ddd, *J* = 8.0, 6.5, 1.1 Hz, 1H), 5.97 (dt, *J* = 8.0, 1.2 Hz, 1H), 4.36 (ddd, *J* = 7.1, 3.6, 1.1 Hz, 1H), 4.18 (dd, *J* = 7.1, 1.1 Hz, 1H), 3.79 (s, 3H), 3.25 (dddd, *J* = 6.4, 3.6, 2.7, 1.3 Hz, 1H), 3.16 (ddd, *J* = 12.0, 10.4, 5.2 Hz, 1H), 3.11 (d, *J* = 2.7 Hz, 1H), 2.85 (dddd, *J =* 15.5, 10.4, 3.8, 0.6 Hz, 1H), 2.72—2.63 (m, 1H), 2.63—2.55 (m, 1H), 1.54 (s, 3H), 1.33 (s, 3H), 1.29 (s, 3H). **^13^C-NMR** (100 MHz, CDCl_3_): δ (ppm) = 174. 0, 136.1, 135.2, 129.1, 127.3, 122.0, 121.5, 119.3, 118.7, 114.0, 111.1, 109.1, 82.3, 74.1, 63.6, 58.2, 52.3, 52.1, 37.8, 25.6, 25.3, 23.8, 19.7. **MS** (EI, 70eV) *m*/*z* (%): 396.5 (4.1, M+), 381.2 (8), 266.2 (100), 144.2 (17),130.1 (17). **HRMS** (ESI+) calculated

**7: ^1^H-NMR** (400 MHz, CDCl_3_): δ (ppm) = 8.06 (s, 1H), 7.60 (dd, J = 7.8, 1.0 Hz, 2H), 7.38 (dt, J = 8.1, 1.0 Hz, 2H), 7.21 (ddd, J = 8.2, 7.0, 1.3 Hz, 2H), 7.14 (ddd, *J* = 8.2, 7.1, 1.1 Hz, 1H), 7.04 (d, J = 2.4 Hz, 1H), 6.14 (dt, J = 8.1, 1.4 Hz, 2H), 6.02 (dd, *J* = 8.1, 1.8 Hz, 1H), 4.39 (ddd, J = 7.2, 3.5, 1.0 Hz, 2H), 4.18 (dd, J = 7.2, 1.1 Hz, 1H), 3.77–3.73 (m, 4H), 3.38 (ddd, *J* = 12.2, 6.8, 3.7 Hz, 1H); 3.30 (ddt, *J* = 5.3, 3.5, 1.9 Hz, 1H); 3.24 (d, *J* = 2.0 Hz, 1H); 2.94—2.85 (m, 1H), 2.85—2.76 (m, 1H), 2.67 (ddd, J = 12.4, 10.9, 5.1 Hz, 1H), 1.56 (s, 4H), 1.34 (s, 4H), 1.29 (s, 3H). **^13^C-NMR** (100 MHz, CDCl_3_): δ (ppm) = 174.4, 138.9, 136.2, 127.4, 127.1, 122.0, 121.8, 119.3, 118.7, 114.1, 111.2, 108.7, 78.4, 63.5, 56.9, 52.3, 51.6, 39.5, 31.0, 26.7, 25.6, 25.3, 20.4.

**(1*S***, **4*S***, **5*R***, **6*S*)-1-Methyl-3-hydroxymethyl-5**,**6-isopropylidendioxy-*N*-tosyl-2-azabicycle[2.2.2]-7-octene(8**, **7:3 exo:endo)**

In a round bottom flask under N_2_ atmosphere, 1.5 equiv. of LiAlH_4_ was dissolved in dry THF (enough volume to get a 0.24 M solution) at 0 °C. A 0.24 M solution of **4** in THF was added dropwise. The reaction was allowed to warm to room temperature. Once the started material was consumed, the reaction mixture was cooled to 0 °C, and ethyl acetate was added (0.5 mL for each mL of the total reaction volume) followed by the addition of the same volume of a 10% aqueous solution of KOH. The mixture was diluted with water and extracted three times with ethyl acetate. The combined organic phases were dried over Na_2_SO_4_ and the solvent was evaporated under reduced pressure, to give alcohol **8**. The crude was sufficiently pure to use in the next reaction, yield = 72%.

**8 *exo* (3*R*): ^1^H-NMR** (400 MHz, CDCl_3_): δ (ppm) = 7.67 (d, *J* = 8.3 Hz, 2H), 7.28 (d, *J* = 8.3 Hz, 2H), 6.02 (dd, *J* = 8.1, 8.1 Hz, 1H), 5.56 (d, *J* = 8.1 Hz, 1H), 4.64 (dd, *J* = 7.1, 3.8 Hz, 1H), 4.10 (dd, *J* = 11.4, 5.2 Hz, 1H), 4.04 (d, *J* = 7.1 Hz, 1H), 3.83 (dd, *J =* 11.4, 6.3 Hz, 1H), 3.52 (ddd, *J* = 6.3, 5.2, 3.3 Hz, 1H), 3.27 (ddd, *J* = 3.8, 3.4, 1.2 Hz, 1H), 1.64 (s, 3H), 1.26 (s, 3H), 1.24 (s, 3H). **^13^C-NMR** (100 MHz, CDCl_3_): δ (ppm) = 143.7, 137.5, 134.7, 130.4, 129.5, 128.1, 127.2, 109.7, 81.3, 72.9, 65.6, 59.8, 37.2, 25.5, 25.3, 21.7, 20.3. **8 *endo* (3*S*): ^1^H-NMR** (400 MHz, CDCl_3_): δ (ppm) = 7.75 (d, *J* = 8.2 Hz, 2H), 7.33—7.29 (m, 2H), 6.22 (7, *J* = 7.1 Hz, 1H), 5.83 (d, *J* = 8.0 Hz, 1H), 4.52 (d, *J* = 7.2 Hz, 1H), 4.38 (d, *J* = 3.2 Hz, 1H), 4.03—4.00 (m, 1H), 3.94 (dd, *J =* 10.6, 5.0 Hz, 1H), 3.58 (dd, *J* = 8.1, 2.6 Hz, 1H), 3.40—3.35 (m, 1H), 2.44 (s, 3H), 1.28 (s, 3H), 1.23 (s, 3H). **^13^C-NMR** (100 MHz, CDCl_3_): δ (ppm) = 143.5, 140.4 135.3, 131.1, 129.7, 127.2, 109.2, 79.0, 76.2, 65.4, 59.2, 57.6, 38.6, 21.6, 21.5, 20.0. **IR** (**8 *exo* + 8 *endo***): ν_max_ (cm^−1^): 3482, 2986, 2934, 1341, 1161, 708.

**(1*S***, **4*S***, **5*R***, **6*S*)-1-Methyl-3-(dimethyl(1**,**1**,**2-trimethylpropyl)silyloxy)methyl-5**,**6-isopropylidendioxy-2-azabicycle[2.2.2]7-octene(9**, **7:3 exo:endo**, **only major isomer is informed):**

To a solution of **8** in DMF at 0 °C under N_2_ atmosphere, imidazole (3 equiv.) and TDSCl (2 equiv.) were added. The reaction was allowed to warm to room temperature and was stirred until total consumption of the starting material. Then, the reaction mixture was diluted with water and extracted three times with Et_2_O. The combined organic phases were washed with a saturated solution of CuSO_4_ and dried over Na_2_SO_4._ The solvent was evaporated under reduced pressure and the crude was purified by SiO_2_ column chromatography using Hex(9):AcOEt(1) as mobile phase, yield = 95%.

9 exo (3S): **^1^H-NMR** (400 MHz, CDCl_3_): δ (ppm) = 7.64 (d, *J* = 8.0 Hz, 2H), 7.25 (d, *J* = 7.7 Hz, 2H), 6.01 (ddd, *J* = 7.8, 6.8, 0.9 Hz, 1H), 5.53 (dt, *J* = 8.1, 1.2 Hz, 1H), 4.67 (dd, *J* = 6.8, 3.9 Hz, 1H), 4.27 (dd, *J* = 10.0, 4.9 Hz, 1H), 4.00 (dd, *J* = 7.0, 1.1 Hz, 1H), 3.53 (t, *J* = 10.0, 1H), 3.46 (ddd, *J* = 10.1, 5.0, 3.1 Hz, 1H), 3.37 (dtd, *J* = 6.7, 3.5, 1.2 Hz, 1H), 1.66 (dq, *J* = 8.9, 6.9 Hz, 1H), 2.4 (s, 3H), 0.92 (s, 3H), 0.93 (s, 3H), 0.91 (s, 3H), 0.90 (s, 3H), 0.89 (s, 6H), 0.15 (s, 6H). **^13^C-NMR** (100 MHz, CDCl_3_) δ (ppm): 143.4, 138.1, 134.7, 130.6, 129.4, 128.1, 109.5, 81.4, 76.7, 73.1, 64.1, 59.2, 58.4, 34.9, 34.2, 25.5, 25.3, 25.1, 21.5, 20.3, 20.3, 20.2, 20.1, 18.6, −1.5, −3.3, −3.6.

**(1*S***, **3*R***, **4*S***, **5*R***, **6*S*)-1-Methyl-3-(dimethyl(1**,**1**,**2-trimethylpropyl)silyloxy)methyl-5**,**6-isopropylidendioxy-2-azabicycle[2.2.2]-7-octene(10 exo) and(1*S***, **3*S***, **4*S***, **5*S***, **6*R*)-1-Methyl-3-(dimethyl(1**,**1**,**2-trimethylpropyl)silyloxy)methyl-5**,**6-isopropylidendioxy-2-azabicycle[2.2.2]-7-octene(10 endo)**

Naphthalene (14 equiv.) was dissolved in dry DME (0.6 mL/mmol of naphthalene) and 11 equiv. of Na was added in portions. The bluish-green reaction mixture was stirred at room temperature for 25 min. Next, this suspension was added dropwise to a 0.1 M solution of **9** in dry DME at −78 °C until persistence of the green color or total consumption of the starting material. Once the reaction was completed, it was allowed to warm to room temperature and a saturated solution of NaHCO_3_ was added. The reaction was extracted three times with ethyl acetate. The organic phases were combined and dried over Na_2_SO_4_. The solvent was evaporated under reduced pressure and the crude was purified by SiO_2_ column chromatography using Hex(8):AcOEt(2) as mobile phase. Compound 10 exo was obtained with a 46% yield and compound 10 endo with a 12%. Full characterization of both compounds was made over their indolic derivatives **11** and **12**, respectively.

**10 exo: ^1^H-NMR** (400 MHz, CDCl_3_): δ (ppm) = 6.31 (ddd, *J* = 7.9, 6.7, 1.0 Hz, 1H), 5.99 (dt, *J* = 8.1, 1.2 Hz, 1H), 4.49 (ddd, *J* = 7.2, 3.5, 1.0 Hz, 1H), 3.92 (dd, J = 7.0, 1.1 Hz, 1H), 3.64 (dd, J = 10.5, 5.5 Hz, 1H), 3.45 (dd, *J* = 10.3, 9.2 Hz, 1H), 3.13 (dddd, *J* = 6.8, 3.5, 2.2, 1.2 Hz, 1H), 2.65 (ddd, J = 9.3, 5.6, 2.2 Hz, 1H), 1.64 (hept, *J* = 6.8; 1H), 1.35 (s, 3H), 1.33 (s, 3H), 1.29 (s, 3H), 0.91 (s, 3H), 0.89 (s, 3H), 0.87 (s, 6H), 0.12 (s, 3H), 0.11 (s, 3H). **^13^C-NMR** (100 MHz, CDCl_3_) δ (ppm) = 134.9, 132.2, 108.2, 82.6, 74.0, 64.8, 55.4, 54.0, 36.1, 34.2, 25.6, 25.2, 25.1, 21.9, 20.3, 20.3, 18.5. **MS** (EI, 70eV) *m*/*z* (%): 69.10 (71), 71.10 (82), 84.10 (68), 98.10 (100), 134.10 (44), 299.25 (9), 367.30 (1).

**10 endo: ^1^H-NMR** (400 MHz, CDCl_3_): δ (ppm)= 5.96—6.04 (m, 2H), 4.32 (ddd, *J* = 7.1, 3.5, 0.9 Hz, 1H), 3.95 (dd, *J* = 7.1, 1.0 Hz, 1H), 3.28 (dd, *J* = 9.5, 6.1 Hz, 1H), 3.19 (dd, *J* = 9.5, 8.2 Hz, 1H), 3.08 (ddt, *J* = 5.4, 3.5, 1.8 Hz, 1H), 2.92 (dd, *J* = 8.2, 6.2 Hz, 1H), 1.59 (q, *J* = 6.8, 6.8 Hz, 1H), 1.31 (s, 6H), 1.28 (s, 3H), 0.86 (s, 3H), 0.85 (s, 3H), 0.81 (s, 6H), 0.04 (s, 6H). **^13^C-NMR** (100 MHz, CDCl_3_) δ (ppm) = 136.7, 128.6, 109.3, 83.4, 78.5, 65.3, 54.8, 53.8, 37.8, 34.6, 31.4, 26.1, 25.6, 25.6, 22.4, 20.8, 20.7, 19.0, 19.0. **IR:** ν_max_ (cm^−1^): 2959; 2938; 2901; 2868; 1099; 1110—1020; 777.

**(1*S***, **3*R***, **4*S***, **5*S***, **6*R*)-*N*-(2-(3-indolyl)ethyl)-1-methyl-3-(dimethyl(1**,**1**,**2-trimethylpropyl)silyloxy)methyl-5**,**6-isopropylidendioxy-2-azabicycle[2.2.2]-7-octene(11)**

This compound was obtained from **10 exo** following the same procedure of coupling with 3-(2-bromoethyl)indole used for compound **6**. The product was purified by column chromatography using Hex(9):AcOEt(1) as mobile phase, yield = 70%.

**^1^H-NMR** (400 MHz, CDCl_3_): δ (ppm) = 7.97 (s, 1H); 7.55 (d, *J* = 7.8 Hz, 1H), 7.35 (dt, *J* = 8.1, 0.9 Hz, 1H), 7.18 (ddd, *J* = 8.2, 7.1, 1.2 Hz, 1H), 7.11 (ddd, *J* = 8.0, 7.0, 1.1 Hz, 1H), 6.95 (d, *J* = 2.3 Hz, 1H), 6.27 (ddd, *J* = 7.8, 6.7, 0.9 Hz, 1H), 5.95 (dt, *J* = 8.0, 1.1 Hz, 1H), 4.53 (dd, *J* = 7.2, 3.5 Hz, 1H), 3.92 (dd, *J* = 7.2, 1.0 Hz, 1H), 3.65 (dd, *J* = 10.5, 5.1 Hz, 1H), 3.41 (t, *J* = 10.3 Hz, 1H), 3.20 (dddd, *J* = 6.6, 3.7, 2.7, 1.2 Hz, 1H), 3.01 (ddd, *J* = 13.3, 11.7, 4.2 Hz, 1H), 2.87 (ddd, *J* = 13.1, 11.2, 4.3 Hz, 1H), 2.74 (ddd, *J* = 13.3, 11.1, 5.0 Hz, 1H), 2.60 (ddd, *J* = 13.3, 11.1, 5.0 Hz, 1H), 2.46 (ddd, *J* = 10.2, 5.1, 2.6 Hz, 1H), 1.58–1.69 (m, 3H), 1.48 (s, 3H), 1.33 (s, 3H), 1.29 (s, 3H), 0.91 (s, 3H), 0.89 (s, 3H), 0.86 (s, 6H), 0.10 (s, 3H). **^13^C-NMR** (100 MHz, CDCl_3_): δ (ppm) = 136.2, 134.5, 130.6, 127.4, 121.0, 121.3, 119.3, 118.7, 114.6, 111.1, 108.7, 82.8, 73.8, 63.9, 61.6, 58.1, 51.9, 34.9, 34.2, 30.9, 25.7, 25.3, 25.1, 24.8, 20.4, 19.8, 18.6. **HRMS** (ESI+) calculated for C_30_H_46_N_2_O_3_Si=510.3278 (M+H) found 511.3350. **IR:** ν_max_ (cm^−1^): 3418, 3350, 2957, 2936, 2868, 1188—1070, 777, 739. **[α]_D_** = −22.4 (c 0.63, MeOH);

**(1*S***, **3*S***, **4*S***, **5*S***, **6*R*)-*N*-(2-(3-indolyl)ethyl)-1-methyl-3-(dimethyl(1**,**1**,**2-trimethylpropyl)silyloxy)methyl-5**,**6-isopropylidendioxy-2-azabicycle[2.2.2]-7-octene (12)**

This compound was obtained from 10 endo following the same procedure of coupling with 3-(2-bromoethyl)indole used for compound **6**. The product was purified by column chromatography using Hex(8):AcOEt(2) as mobile phase, yield = 72%.

**^1^H-NMR** (400 MHz, CDCl_3_): δ (ppm) = 7.98 (s, 1H), 7.55 (d, *J* = 7.9 Hz, 1H), 7.37 (dt, *J* = 8.1, 0.9 Hz, 1H), 7.20 (ddd, *J* = 8.2, 7.0, 1.2, Hz, 1H), 7.13 (ddd, *J* = 8.0, 7.0, 1.0 Hz, 1H), 7.01 (d, *J* = 2.3 Hz, 1H), 6.06 (dd, *J* = 8.1, 6.1, Hz, 1H), 6.00 (ddd, *J* = 8.1, 1.8, 1.0 Hz, 1H), 4.32 (ddd, *J* = 7.2, 3.5, 0.7 Hz, 1H), 4.23 (dd, *J* = 7.2, 1.0 Hz, 1H), 3.51 (dd, *J* = 9.8, 4.2 Hz, 1H), 3.33—3.23 (m, 3H), 2.98—2.80 (m, 2H), 2.61 (ddd, *J* = 12.5, 10.6, 6.0 Hz, 1H), 2.56—2.50 (m, 1H), 1.68—1.55 (m, 3H), 1.49 (s, 3H), 1.33 (s, 3H), 1.26 (s, 3H), 0.90 (s, 3H), 0.88 (s, 3H), 0.85 (s, 3H), 0.84 (s, 3H), 0.08 (s, 3H), 0.07 (s, 3H). **^13^C-NMR** (100 MHz, CDCl_3_): δ (ppm) = 137.8, 136.2, 129.6, 127.4, 122.1, 121.5, 119.4, 118.7, 114.3, 111.2, 108.1, 78.6, 65.6, 63.5, 57.0, 51.9, 37.5, 34.2, 28.3, 25.6, 25.2, 25.1, 20.7, 20.4, 20.4, 18.6, −3.3, −3.3. **IR:** ν_max_ (cm^−1^): 3421, 3345, 2957, 2926, 2868, 1099—1061, 739. **HRMS** (ESI+) calculated for C_30_H_46_N_2_O_3_Si = 510.3278 (M+H) found 511.3355. **[α]_D_** = 18.4 (c 0.25, CH_2_Cl_2_).

**(1*S***, **3*R***, **4*S***, **5*S***, **6*R*)-*N*-(2-(3-indolyl)ethyl)-1-methyl-3-hydroxymethyl-5**,**6-isopropylidendioxy-2-azabicycle[2.2.2]-7-octene (13)**

To a 0.01M solution of **11** in dry THF, TBAF (5 equiv.) was added, and the reaction mixture was stirred at room temperature until total consumption of the starting material. The crude was diluted at half with ethyl acetate and washed with a saturated solution of NH_4_Cl and brine. The organic phase was dried over Na_2_SO_4,_ and the solvent was evaporated under reduced pressure_._ The crude was filtered through a pad of SiO_2_ and eluted with Hex(6):AcOEt(4), yield = 80%.

**^1^H-NMR** (400 MHz, CDCl_3_): δ (ppm) = 8.03 (s, 1H), 7.53 (dq, *J* = 7.8, 0.9 Hz, 1H), 7.37 (dt, *J* = 8.1, 1.0 Hz, 1H), 7.22 (ddd, *J* = 8.2, 7.0, 1.2 Hz, 1H), 7.13 (ddd, *J* = 8.0, 7.0, 1.1 Hz, 1H), 6.99 (d, *J* = 2.4 Hz,1H), 6.30 (ddd, *J* = 7.9, 6.5, 1.0 Hz, 1H), 5.99 (dt; *J* = 7.9, 1.0 Hz, 1H), 4.72 (ddd, *J* = 7.1, 3.8, 0.9 Hz, 1H), 3.93 (dd, *J* = 7.1, 1.0 Hz, 1H), 3.64 (dd, *J* = 10.9, 6.3 Hz, 1H), 3.56 (dd, *J* = 10.8, 2.2 Hz, 1H), 3.15—3.06 (m, 1H), 2.95 (dddd, *J* = 6.5, 3.8, 2.7, 1.2 Hz, 1H), 2.93—2.86 (m, 1H), 2.81—2.66 (m, 2H), 2.55 (dt, *J* = 6.3, 2.5 Hz, 1H), 1.52 (s, 3H), 1.31 (s, 3H), 1.27 (s, 3H). **^13^C-NMR** (101 MHz, CDCl_3_) δ (ppm) = 136.3, 134.1, 130.8, 127.3, 122.1, 121.4, 119.3, 118.6, 114.1, 111.2, 108.9, 83.1, 73.9, 62.0, 60.8, 58.6, 51.5, 39.7, 25.6, 25.3, 24.8, 20.0. **HRMS** (ESI+) calculated for C_22_H_28_N_2_O_3_=368.2100 (M+H) found 369.2173. **IR** ν_max_ (cm^−1^): 3419, 1640, 1000—1085, 741, 708. **[α]_D_** = −19.1 (c 0.11, MeCN);

**(1*S***, **3*R***, **4*S***, **5*S***, **6*R*)-*N*-(2-(3-indolyl)ethyl)-1-methyl-3-benzyloximethyl-5**,**6-isopropylidendioxy-2-azabicycle[2.2.2]-7-octene (14)**

A 0.02M solution of **13** in DMF was cooled to 0 °C under nitrogen atmosphere and NaH (2 equiv.) was added. The reaction mixture was stirred for 5 min and BnBr (4 equiv.) was added. Next, the solution was allowed to warm to room temperature and stirred until total consumption of the starting material. The reaction was finished with the addition of a saturated solution of NH_4_Cl, and it was extracted three times with Et_2_O. The combined organic phases were washed with a saturated solution of CuSO_4_ and brine. The organic phase was dried over Na_2_SO_4_ and the solvent was evaporated under reduced pressure. The crude was purified by SiO_2_ column chromatography using Hex(9):AcOEt(1) as mobile phase, yield = 50%.

**^1^H-NMR** (400 MHz, CDCl_3_): δ (ppm) = 7.97 (s, 1H), 7.57 (d, *J* = 7.8), 7.29—7.39 (m, 6H), 7.21 (ddd, *J* = 8.2, 7.0, 1.2 Hz, 1H), 7.13 (ddd, *J* = 8.1, 7.1, 1.1 Hz; 1H), 6.95 (d, *J* = 2.3), 6.28 (ddd, *J* = 8.0, 6.8, 1.1 Hz, 1H), 5.97 (dt, *J* = 8.0, 1.1 Hz, 1H), 4.61 (d, *J* = 11.9 Hz, 1H), 4.53 (d, *J* = 11.9 Hz, 1H), 4.45 (dd, *J* = 7.2, 3.6 Hz, 1H), 3.93 (dd, *J* = 7.2, 1.0 Hz, 1H), 3.58 (dd, *J* = 9.6, 5.3 Hz, 1H), 3.39 (t, *J* = 9.6 Hz, 1H), 3.23 (ddq, *J* = 4.8, 3.7, 1.3 Hz, 2H), 3.07 (ddd, *J* = 13.3, 12.0, 4.1 Hz, 1H), 2.88 (ddd, *J* = 13.3, 11.2, 4.1 Hz, 1H), 2.78 (ddd, *J* = 13.4, 11.2, 4.7 Hz, 1H), 2.68 (ddd, *J* = 9.5, 5.1, 2.1 Hz, 1H), 1.51 (s, 3H), 1.35 (s, 3H), 1.28 (s, 3H). **^13^C-NMR** (100 MHz, CDCl_3_): δ (ppm) = 138.2, 136.2, 134.5, 130.4, 128.4, 127.8, 127.7, 127.4, 122.0, 121.3, 119.3, 118.8, 114.5, 111.1, 108.7, 82.7, 73.8, 73.3, 71.6, 59.3, 58.0, 51.8, 35.6, 29.7, 25.7, 25.3, 24.7, 19.8. **HRMS** (ESI+) calculated for C_29_H_34_N_2_O_3_ = 458.2569 (M+H) found 459.2642 **IR:** ν_max_ (cm^−1^): 2940, 1447, 1242, 1047, 1098, 737. **[α]_D_** = 133 (c 0.23, MeCN);

**(1*S***, **3*R***, **4*S***, **5*S***, **6*R*)-*N*-(2-(3-indolyl)ethyl)-1-methyl-3-(2-(1*H*-3-indolyl)acetyloxy)methyl-5**,**6-isopropylidendioxy-2-azabicycle[2.2.2]-7-octene (15)**

Alcohol **13** was added to a 0.08M solution of 3-indoleacetic acid (1 equiv.) in dry CH_2_Cl_2_. Next, HBTU (1.2 equiv.), dry DIPEA (1.2 equiv.) and DMAP (0.2 equiv.) were added. The solution was stirred for 24 h at room temperature, and once the reaction was completed, the crude was filtered through Celite. The solvent was evaporated under reduced pressure, the residue was redissolved in AcOEt, washed with a 5% aqueous solution of HCl and then with a saturated solution of NaHCO_3_. The organic phase was dried over NaSO_4_, filtered, and the solvent was evaporated under reduced pressure. The crude was purified by SiO_2_ column chromatography using Hex(1):AcOEt(1) as mobile phase, yield = 87%.

**^1^H-NMR** (400 MHz, CDCl_3_): δ (ppm) = 8.07 (s, 1H), 7.98 (s, 1H), 7.64 (ddd, *J* = 7.9, 2.1, 0.9 Hz, 1H), 7.59 (ddd, *J* = 7.9, 1.8, 0.9 Hz, 1H), 7.36 (dt, *J* = 6.0, 1.1 Hz, 1H), 7.34 (dt, *J* = 5.6, 1.1 Hz, 1H), 7.24—7.28 (m, 2H), 7.18–7.13 (m, 2H), 7.12–7.11 (m, 1H), 6.93 (d, *J* = 2.2, 1H); 6.20 (ddd, *J* = 7.8, 6.6, 0.9 Hz; 1H), 5.95 (dt, *J* = 8.1, 1.2 Hz, 1H), 4.52 (dd, *J* = 7.2, 3.5 Hz, 1H), 4.27 (dd, *J* = 11.4, 5.6 Hz, 1H), 4.02 (dd, *J* = 11.4, 9.2 Hz, 1H), 3.94 (d, *J* = 7.1 Hz, 1H), 3.06 (ddd, *J* = 13.4, 11.9, 4.1 Hz, 1H), 2.97 (dddd, *J* = 6.5, 3.7, 2.6, 1.2 Hz, 1H), 2.91—2.82 (m, 1H); 2.77 (ddd, *J* = 13.5, 11.2, 4.6 Hz, 1H), 2.70—2.60 (m, 2H), 1.50 (s, 3H), 1.31 (s, 3H), 1.26 (s, 3H). **^13^C-NMR** (100 MHz, CDCl_3_) δ (ppm) = 171.9, 136.2, 136.1, 134.9, 129.9, 127.4, 127.2, 123.1, 122.2, 122.0, 121.4, 119.7, 119.3, 118.8, 118.8, 114.4., 111.2, 111.1, 108.2, 82.6, 73.7, 65.6, 58.2, 58.1, 51.1, 35.7, 31.4, 25.7, 25.3, 24.5, 19.7. **HRMS** (ESI+) calculated for C_32_H_35_N_3_O_4_ = 525.2628 (M+H) found 526.2701 **IR:** ν_max_ (cm^−1^): 3412, 2096, 1645, 1456, 1373, 1265, 1163, 1095–987, 738. **[α]_D_** = 15.7 (c 0.34, CH_2_Cl_2_).

### 4.2. GDNF Release and Toxicity Evaluation

*GDNF release by C6 cells:*

Cell culture: Rat C6 glioma cells were purchased from the American Type Culture Collection (CC-107) and maintained in Dulbecco’s Modified Eagle Medium (Life Technologies, Carlsbad, CA, USA; 10569) with 5% (*v*/*v*) fetal bovine serum (FBS, Atlanta Biologicals) and 100 U mL−1 of penicillin and streptomycin (Life Technologies). Cells were incubated at 37 °C with 5% CO_2_ humidified atmosphere. As a glioma cell line, C6 cells are highly susceptible to phenotypic drift, which can lead to varying expression levels of receptors and growth factors of interest. For such reason, all data presented here show a single representative experiment of many independent replicate trials. C6 glioma cells were used between passages 41−42, where they showed low variability [15].

GDNF release experiments: Into a 96-well plate were added C6 cells at a density of 25,300 cells/well in full growth medium (see above). Cells were allowed to adhere for 24 h at 37 °C. Cells were then serum-starved with media containing 0.5% FBS (low serum) for an additional 24 h. Low-serum media was refreshed prior to starting the experiment. Compounds were added in 50 μL of low-serum media to obtain a final volume of 200 μL/well. Treatments were performed in quadruplicate. Cells were incubated at 37 °C for 24 h. Experiments were terminated by removing the conditioned media from each well and storing them at −80 °C until analyzed. GDNF was detected using a standard sandwich-style ELISA kit purchased from Promega Corporation (Madison, WI, USA) following the manufacturer’s instructions. Briefly, monoclonal anti-GDNF antibodies were captured onto a 96-well Nunc Immulon Immunoassay plate at a dilution of 1:1000 in carbonate coating buffer (25 mM sodium bicarbonate, 25 mM sodium carbonate, pH 8.2) overnight at 4 °C. After removing the monoclonal antibody, wells were blocked with 1× block and sample buffer for 1 h at RT (200 μL/well). A GDNF standard curve was created by serially diluting the recombinant human GDNF standard in 1× block and sample buffer to a concentration range of 0−1000 pg mL^−1^. To each sample well was added 100 μL of conditioned media from above and the standard curve (in duplicate), and plates were incubated for 6 h with shaking at RT. After washing five times with TBST (150 mM sodium chloride, 10 mM Tris·HCl, 10 mM Tris base, 0.05% Tween 20, pH 7.6), wells were incubated with antihuman polyclonal GDNF antibodies (1:500) in 1× block and sample buffer overnight at 4 °C. Following an additional five washes with TBST, wells were incubated with antichicken IgY-HRP conjugate antibody (1:250) for 2 h with light shaking. After a final five washes, TMB One (100 μL/well) was added to each well and allowed to develop in the absence of light until there were clear differences in color between the highest and lowest concentrations of the standard curve. Wells were then quenched with 1 M HCl (100 μL/well), and the plates were read at an absorbance wavelength of 450 nm using a BioTek Synergy H1 plate reader.

LDH Cytotoxicity Assay: The lactate dehydrogenase cytotoxicity assay (Promega) was performed following the manufacturer’s instructions. Briefly, following compound treatment, conditioned media were removed, and untreated wells were washed twice with phosphate buffered saline. To untreated wells was added 40 μL of low-serum media supplemented with lysis buffer provided in the kit (1:10), protease inhibitor cocktail (1:100), and phosphatase inhibitor cocktail 2 (1:100). Cells were lysed at 37 °C for 1 h. Cell lysates were diluted with 160 μL of conditioned media and used as 100% cytotoxicity in the LDH standard curve. Lysates were serially diluted down to 6.25% cytotoxicity with low-serum media filling the last. The standard curve was added in duplicate to a 96-well plate followed by the conditioned media from each treated well at 50 μL/well. To each well was added 50 μL of the reconstituted substrate mix, and the plates were allowed to develop in the dark until differences were seen in the standard curve. The wells were quenched with 50 μL of stop solution, and the plates were read at an absorbance wavelength of 490 nm.

WST-1 Cell Viability Assay: After compound treatment, conditioned media were removed and replaced with 75 μL of warm low-serum media. To each well was added 5 μL of WST-1 Cell Proliferation Reagent (Roche Applied Science, Penzberg, Germany), and the cells were incubated at 37 °C for no more than 1 h. Plates were briefly shaken prior to reading the absorbance at 450 nm. Treatments were compared to vehicle control.

Statistical Analysis: Data analysis was performed using Graphpad Prism 6 Software (San Diego, CA, USA). Conditions are expressed as mean ± SD and were subjected to ANOVA followed by either Dunnett’s or Tukey’s multiple comparisons test with a significance level of *p* < 0.05.

### 4.3. Viability Assays for T. brucei and Murine Macrophages

The anti-trypanosomal activity of compounds was evaluated against the bioluminescent cell line of the bloodstream stage of *Trypanosoma brucei brucei* (called LUC cell line) as previously described [33]. Briefly, to a 96-well culture plate containing 2.2 μL/well DMSO (negative control) or compounds dissolved in DMSO (1% final concentration), 220 μL/well of a suspension of 1 × 10^5^ parasites/mL was added. The plates were incubated at 37 °C and 5% CO_2_ for 24 h. Next, each well was transferred to a 96-well black plate and 20 μL of a solution containing D-Luciferin (1.5 mg/mL in PBS glucose 1% *w*/*v*) and Triton X-100 (0.05% vol/vol) was added. Bioluminescence signal was measured in a LUMIstar OPTIMA Microplate luminometer using the following settings: 10 s shaking, 5 s/well acquisition, 0.2 s measurement delay, maximum gain, and 37 °C. For the bioluminescence assay, parasite viability was calculated according to the following formula: Viability (%) = (BLcpd—BLblank)/ (Blneg—BLblank) × 100, where BL refers to the mean of bioluminescence signal corresponding to the tested compound (cpd), the blank (blank, complete media containing 1% *v*/*v* DMSO), or the negative control (neg, parasites treated with 1% *v*/*v* DMSO). EC50 values were determined from concentration-response curves fitted to a four-parameter sigmoid equation using the GraphPad Prism software (version 6.0). All errors are expressed as one SD.

Cytotoxicity assays on murine macrophages. Mouse macrophages from the cell line J774 (ATCC^®^ TIB-67TM) were cultivated under a humidified 5% CO_2_/95% air atmosphere at 37 °C in Dulbecco’s modified Eagle’s medium (DMEM) supplemented with 10% (*v*/*v*) FBS, 10 U/mL penicillin and 10 μg/mL streptomycin. The experimental protocol for the determination of EC50 values was essentially the same as that previously described, except that 200 μL/well of a cell suspension at 6 × 10^4^ cells per mL was added in a 96-well culture plate and washes were made with 150 μL of DMEM [36]. The EC50 against macrophages was assayed only for compounds for which the corresponding EC50 towards parasites was previously determined. The cytotoxicity of the compounds was evaluated in triplicate using the WST-1 reagent (Roche). The control treatment included cells cultured in the presence of DMSO 1% (*v*/*v*). Absorbance at 450 nm, corresponding to the formazan dye produced by metabolically active cells, was measured with an EL 800 microplate reader. The corrected absorbance values at 450 nm were obtained by subtracting the corresponding absorbance value at 630 nm and the blank average (e.g., Ai ^c450nm^ = Ai ^450^ − Ai ^630nm^ − Ablank ^450nm^). EC50 values were obtained from drug-response curves as described above for the *T. brucei* assay and the associated errors are expressed as S.D.

## 5. Conclusions

In summary, we report a novel short chemoenzymatic and stereoselective synthesis of six *N*-indolylethyl-substituted isoquinuclidines. The activity of the compounds to increase GDNF release on glioma cells and to inhibit the proliferation of pathogenic trypanosomes was tested. Four compounds displayed GDNF release and anti-trypanosomal activities. One of the compounds, **11**, showed a promising GDNF-releasing activity and further structural modifications are in course to lower its cytotoxicity and increase its GDNF bioactivity. On the other hand, compound **14** emerged as a new head of series for the development of novel derivatives with improved potency and selectivity towards pathogenic trypanosomatids.

## Figures and Tables

**Table 1:** In vitro activity of iboga-like compounds against bloodstream *T. b. brucei*.

Compound	Viability at 10 µM (%)	EC50 (µM) ^a^	SI ^b^
**6**	24 ± 6	7.96 ± 1.71	>12
**7**	42 ± 14	31.52 ± 1.18	ND
**10exo**	64 ± 2	ND	ND
**11**	37 ± 6	7.95 ± 0.34	3.9
**14**	22 ± 12	1.27 ± 0.26	29.6
**15**	0 ± 1	3.47 ± 0.02	5.7
**XL-008**	ND	19.5 ± 0.30	ND
**Nfx**	ND	5.31 ± 0.44 ^c^	ND

ND, not determined. ^a^ The values are expressed as mean SD (n = 3). ^b^ Ratio EC50 murine macrophage/EC50 *T. b. brucei*. ^c^ EC50 reported in Benítez et al. 2020 using the same cell line and assay conditions.

## Data Availability

Not applicable.

## Supplementary Materials

The following are available online, Figure S1: (A) Dose-response curve for compound **6** after 48 h treatment. (B) Effect on cell viability determined by WST-1 assay for compound **6**. (C) Cell lysis determined by LDH assay for compound **6**. Statistics: data represent mean ± SEM of 3 biological replicates. One-way ANOVA followed by Dunnett’s Multiple Comparisons Test is shown (* *p* < 0.05), Figure S2: (A) Dose-response curve after 48 h treatment for compound **14**. (B) Effect on cell lysis determined by LDH assay for compound **14**. (C) Effect on cell viabily determined by WST-1 assay for compound 14, Figure S3: Dose-response curves of compounds **7**, **10**, **11**, **13**, **14** and **XL-008** to determine EC50 against bioluminescent cell line of bloodstream *T. brucei brucei*, Table S1: Optimization of tosyl group removal. a Yield estimated by 1H-NMR of the reaction crude using trichloroethylene as internal standard, Table S2: Optimization of the coupling reaction with the 3-(2-bromoethyl)indoleacetic acid.a Yield estimated by 1H-NMR of the reaction crude using trichloroethylene as internal standard. Reaction was optimized using compound **5a** (Figure S1), obtained by reaction of 4 with Na/Napthalene.

## Sample Availability

Samples of the compounds are available from the authors.
