# Supplementary material for: Iboga Inspired N-Indolylethyl-Substituted Isoquinuclidines as a Bioactive Scaffold: Chemoenzymatic Synthesis and Characterization as GDNF Releasers and Antitrypanosoma Agents
Source: Molecules. 2022 Jan 27;27(3):829. doi: 10.3390/molecules27030829 (PMC8839081; doi:10.3390/molecules27030829)
Supplement: Supplementary file 1 [file molecules-27-00829-s001.zip › molecules-1537304-supplementary.pdf]

Iboga Inspired N-Indolylethyl-Substituted Isoquinuclidines as a Bioactive Scaffold: Chemoenzymatic Synthesis and Characterization as GDNF Releasers and Antitrypanosoma Agents

Supporting Information

Mariana Pazos [1], Estefania Dibello [1,2], Juan Manuel Mesa [1], Dalibor Sames [3], Marcelo Alberto Comini [2], Gustavo Seoane [1] and Ignacio Carrera [1,*]

[1] Laboratorio de Síntesis Orgánica, Departamento de Química Orgánica, Facultad de Química, Universidad de la República, General Flores 2124, Montevideo 11800, Uruguay; mpazos@fq.edu.uy (M.P.); edibello@fq.edu.uy (E.D.); jmmesa@fq.edu.uy (J.M.M.); gseoane@fq.edu.uy (G.S.)
[2] Group Redox Biology of Trypanosomes, Institut Pasteur de Montevideo, Mataojo 2020, Montevideo 11400, Uruguay; mcomini@pasteur.edu.uy
[3] Department of Chemistry, Columbia University, New York, NY 10027, USA; ds584@columbia.edu
* Correspondence: icarrera@fq.edu.uy; Tel.: +598-2-9247-881

Table of Contents

1. Supplementary figures and tables……………………………………………………….2
2. General considerations…………………………………………………………………...5
3. Compound characterization data……………………………………………………..6

1. Supplementary figures and tables

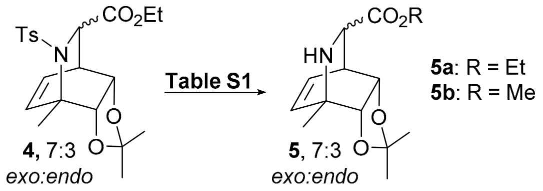

Nº	Reactants	Solvent	T (ºC)	Prod. (%)
1	Na / Naphtalene	DME	-78	**5a** (25%)
2	Mg/)))	MeOH	25	**5b** (93%)[a]
3	Mg/Ti(O-*i*Pr)$_4$/TMSCl	THF	50	Recovery of **4**
4	TMSCl/NaI	MeCN	0 a 85	Decomposition
5	Mg /)))	EtOH	25	Recovery of **4**

Table S1. Optimization of tosyl group removal. [a] Yield estimated by ^{1}H-NMR of the reaction crude using trichloroethylene as internal standard.

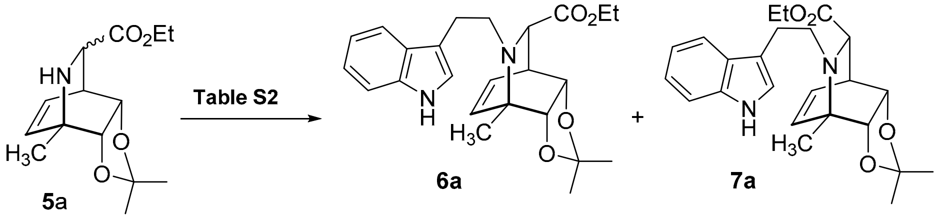

Nº	Base	[Amine] (M)	Time (h)	Yield 6+7 (%)
**1**	$NaHCO_3$	0.02	48	30
**2**	DIPEA	0.25	48	33[a]
**3**	DBU	0.25	48	-
**4**	K_2CO_3	0.25	48	Traces
**5**	K_2CO_3 + 18-c-6	0.25	48	14[a]
**6**	$NaHCO_3$	0.25	18	43

Table S2. Optimization of the coupling reaction with the 3-(2-bromoethyl)indoleacetic acid.[a] Yield estimated by ^{1}H-NMR of the reaction crude using trichloroethylene as internal standard. Reaction was optimized using compound **5a** (**Figure S1**), obtained by reaction of **4** with Na/Napthalene.

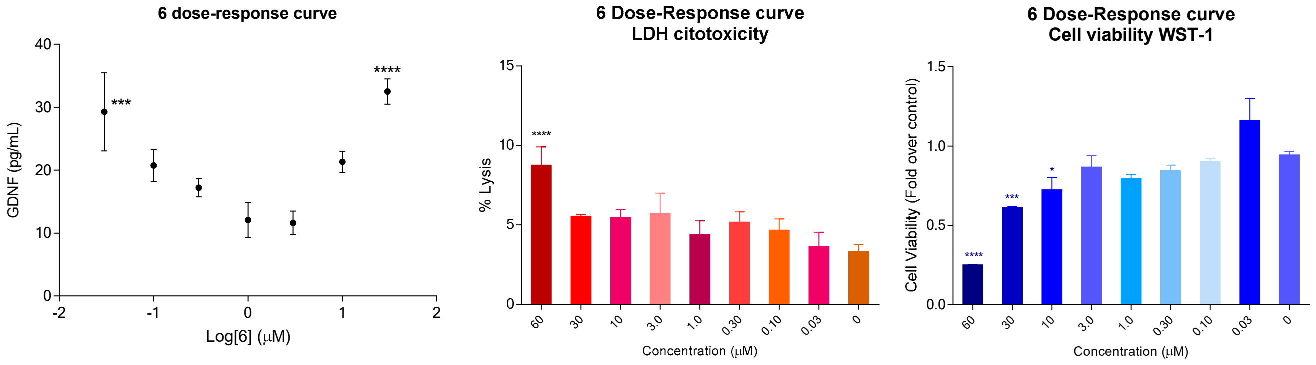

Figure S1 A. Dose-response curve for compound 6 after 48h treatment. **B.** effect on cell viability determined by WST-1 assay for compound 6. **C.** Cell lysis determined by LDH assay for compound 6. **Statistics:** data represent mean ± SEM of 3 biological replicates. One-way ANOVA followed by Dunnett's Multiple Comparisons Test is shown (*p < 0.05).

.

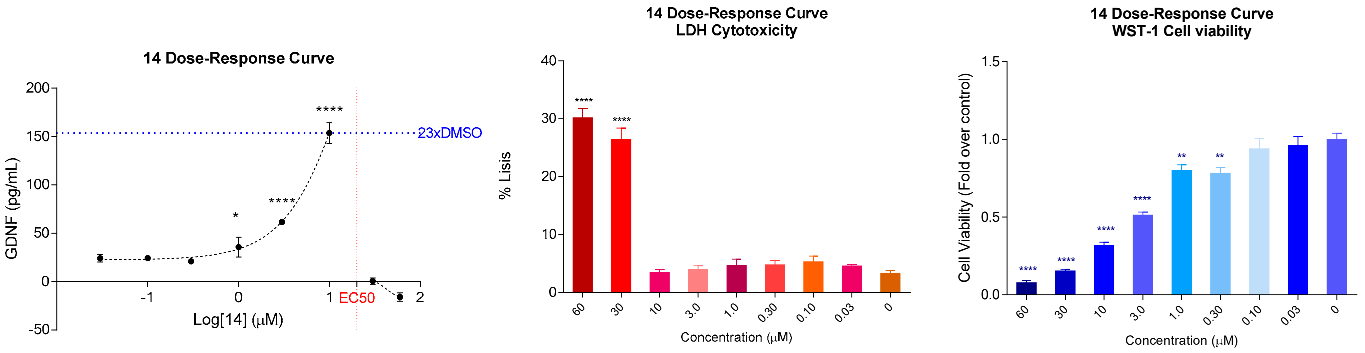

Figure S2 A. Dose-response curve after 48h treatment for compound 14. **B.** Effect on cell lysis determined by LDH assay for compound 14. **C.** Effect on cell viabily determined by WST-1 assay for compound 14.

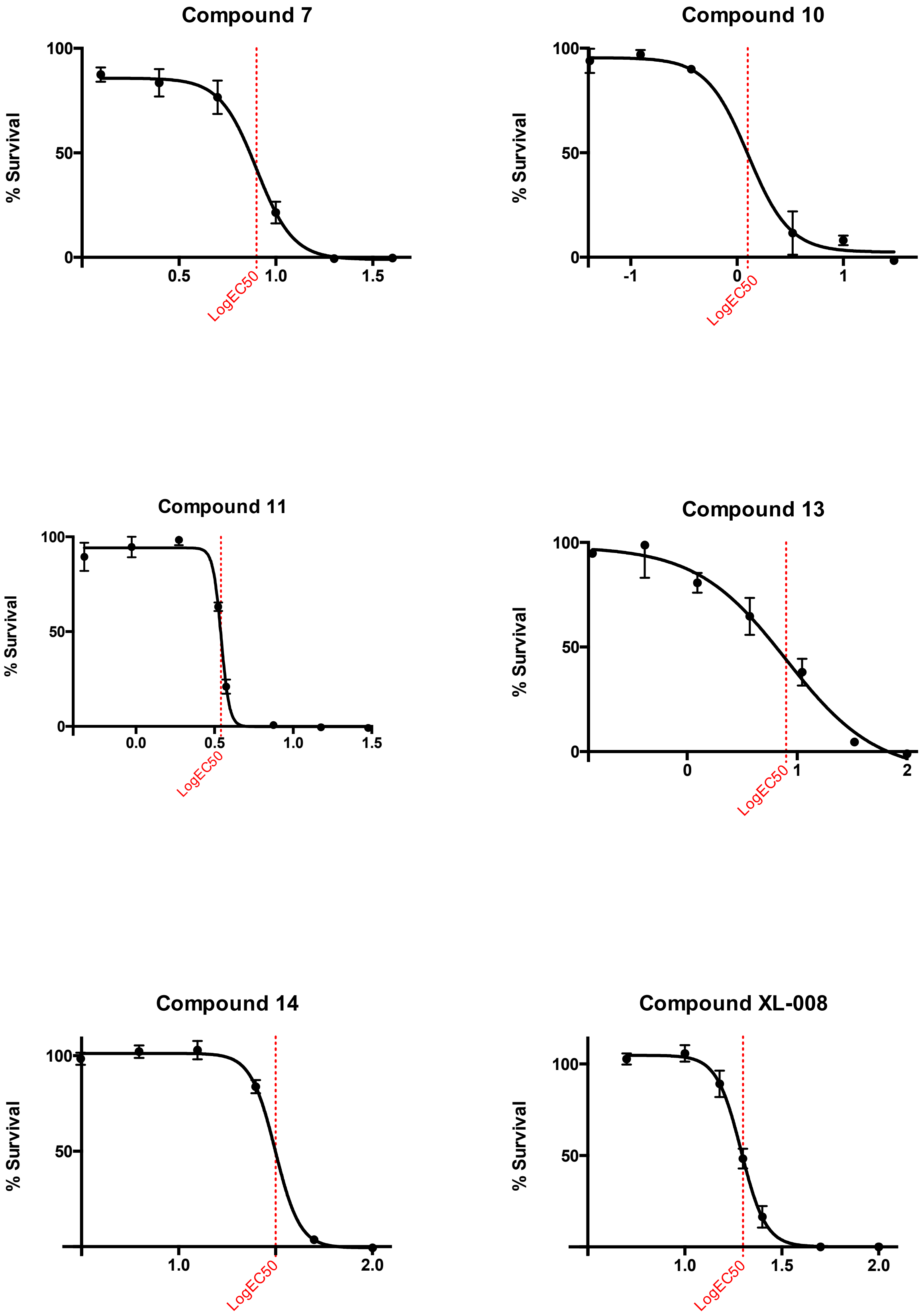

Figure S3. Dose-response curves of compounds **7**, **10**, **11**, **13**, **14** and **XL-008** to determine EC50 against bioluminescent cell line of bloodstream *T. brucei brucei*.

2. General considerations

Chemicals and reagents were purchased from Sigma-Aldrich and used as received. All solvents were distilled prior to use. NMR spectra were obtained in $CDCl_3$ on a Bruker Avance DPX-400 instrument.

Proton chemical shifts (δ) are reported in ppm downfield from TMS as an internal reference, and carbon chemical shifts are reported in ppm relative to the center line of the $CDCl_3$ triplet (77.0 ppm). Optical rotations were measured on a Zuzi 412 polarimeter using a 0.5 dm cell and on a Dichrom P-2000 polarimeter using a 3,5mm x 100mm cell. $[\alpha]_D$ values are given in units of deg.cm^2.g^{-1} and concentration values are expressed in g/100 mL.

High resolution mass spectra were obtained on a Bruker Daltonics Q-TOF spectrometer (ESI mode) and on a Thermo Scientific Q Exactive Plus. Infrared spectra (IR) were recorded either on neat samples (KBr or NaCl disks) or in solution on a Shimadzu FT-IR 8101A spectrophotometer. Analytical TLC was performed on silica gel 60F-254 plates and visualized with UV light (254 nm) and/or p-anisaldehyde in acidic ethanolic solution.

Flash column chromatography was performed using silica gel (Kieselgel 60, EM reagent, 230–400 mesh).

3. Compound characterization data

(1S,4S,7R,8S)-Ethyl-7,8-isopropylidendioxy-1-methyl-2-tosyl-2-azabicycle[2.2.2]5 octenen-3-carboxylate (4, 7:3 exo:endo)

4 **exo :** **^{1}H-RMN** (400 MHz, $CDCl_3$): δ (ppm)= 7.96 (dt, *J* = 1.9, 8.3 Hz, 2H), 7.30 (d, *J* = 8.2 Hz, 2H); 6.22 ddd, *J* = 0.9, 6.7, 8.0 Hz, 1H), 5.96 (dt, *J* = 1.1, 8.1 Hz, 1H), 4.36 (ddd, *J* = 0.7, 3.5, 7.1 Hz, 1H), 4.31 (q, *J* = 7.2 Hz, 2H), 4.24 (d, *J* = 3.3 Hz, 1H), 4.16 (dd, *J* = 0.9, 7.1 Hz, 1H), 3.40 (dddd, *J* = 1.2, 3.4, 3.4, 6.7 Hz, 1H), 2.42 (s, 3H), 1.44 (s, 3H), 1.35 (t, *J* = 7.2 Hz, 3H), 1.25 (s, 3H), 1,23 (s, 3H); **^{13}C-RMN** (100 MHz, $CDCl_3$): δ (ppm) = 170.7, 135.7, 129.5, 128.0, 110.0, 81.0, 73.6, 61.7, 58.9, 58.7, 37.6, 25.5, 25.3, 21.6, 19.2, 14,2; **4 endo :** **^{1}H-RMN** (400 MHz, $CDCl_3$): δ (ppm)= 8.09 (dt, *J* = 1.9, 8.3 Hz, 2H), 7.30 – 7.28 (m, 2H); 6.07 (ddd, *J* = 0.9, 6.4, 7.6 Hz, 1H), 5.91 (ddd, *J* = 1.0, 1.6, 8.0 Hz, 1H), 4.60 (d, *J* = 2.4 Hz, 1H), 4.58 (dd, *J* = 1.0, 7.1 Hz, 1H), 4.53 (ddd, *J* = 1.0, 3.3, 7.1 Hz, 1H), 4.25 – 4.23 (dd, *J* = 0.9, 7.1 Hz, 1H), 3.52 – 4.48 (m, 1H), 2.42 (s, 3H), 1.39 (s, 3H) 1.32 – 1.27 (m, 9H); **^{13}C-RMN** (100 MHz, $CDCl_3$): δ (ppm)= 170.72 , 143.49 , 140.28 , 136.43 , 129.56 , 129.46 , 128.13 , 109.64 , 79.07 , 76.04 , 61.48 , 58.78 , 56.27 , 39.75 , 29.69 , 26.90 , 25.48 , 22.66 , 19.23.

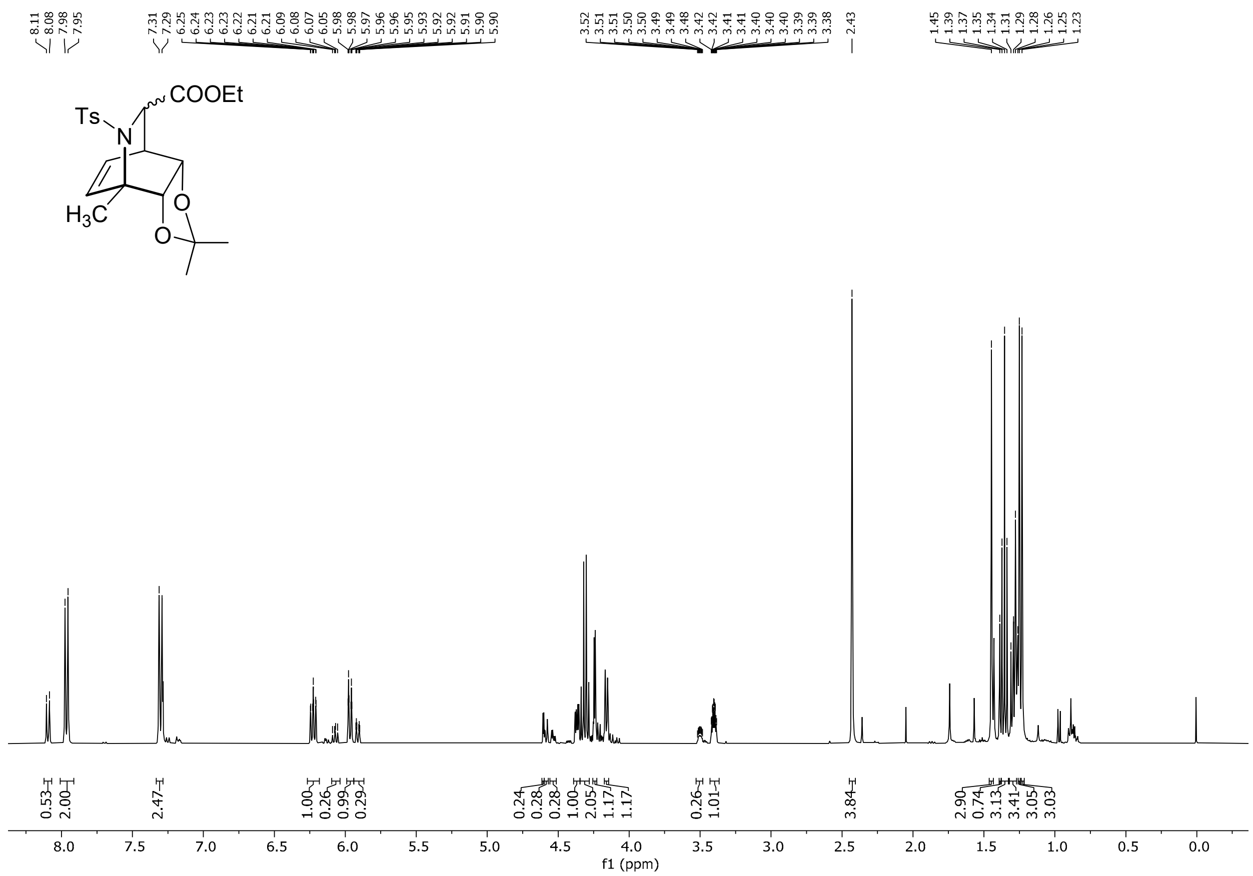

Figure S4. ^{1}H NMR of **4** in $CDCl_3$

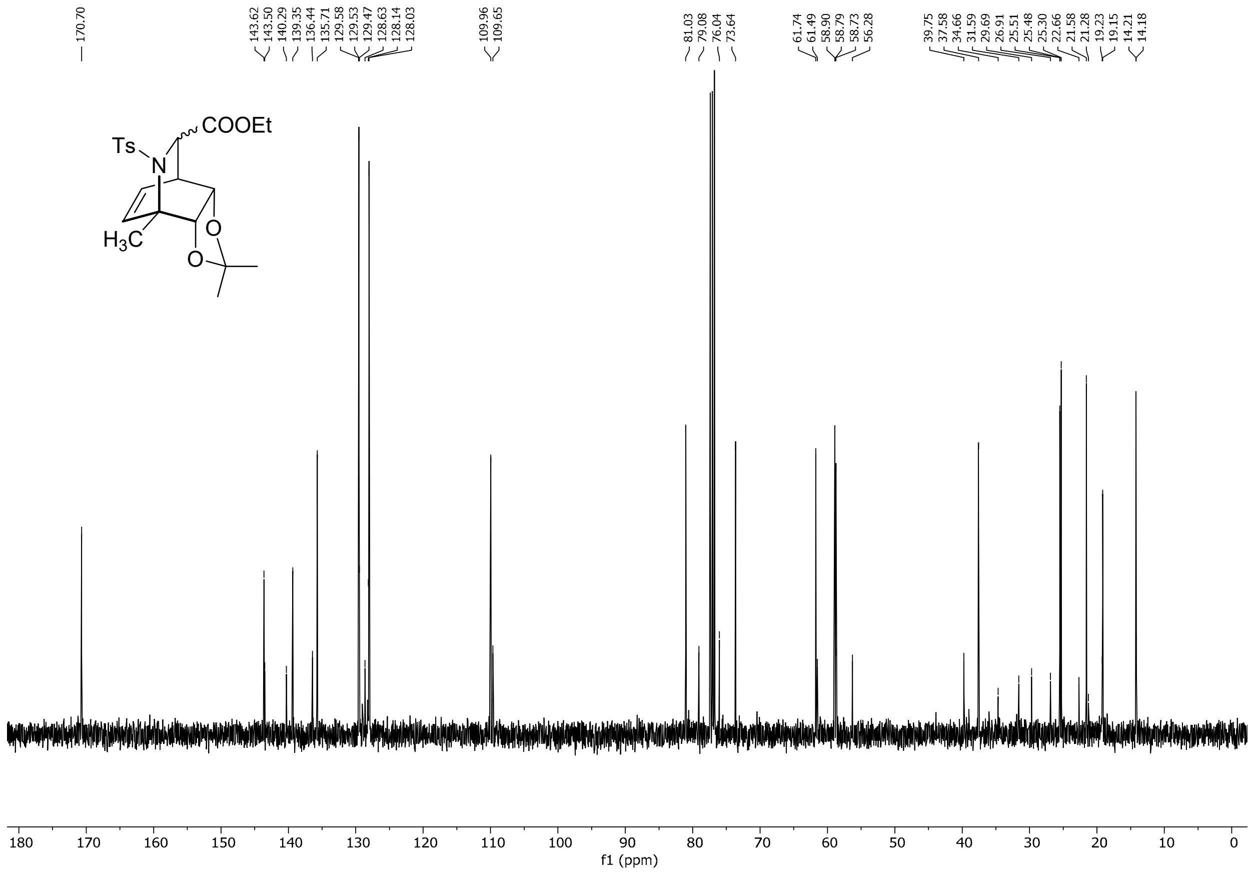

Figure S5. ^{1}H NMR of **4** in $CDCl_3$

(1S , 3R, 4S, 5R, 6S)Methyl-N-(2-(3-indolyl)ethyl)-1-methyl-5,6-isopropylidendioxy-2-azabicycle[2.2.2]-7-octenen-3-carboxylate (6)

^{1}H-RMN (400 MHz, $CDCl_3$): δ (ppm)= 8.01 (s, 1H), 7.57 (ddd, *J* = 8.0, 2.0, 0.9 Hz, 1H), 7.37 (dt, *J* = 8.0, 1.0 Hz, 1H), 7.20 (ddd, *J* = 8.2, 7.0, 1.2 Hz, 1H), 7.13 (ddd, *J* = 8.0, 7.0, 1.2 Hz, 1H), 7.00 (d, *J* = 2.4 Hz, 1H), 6.29 (ddd, *J* = 8.0, 6.5, 1.1 Hz, 1H), 5.97 (dt, *J* = 8.0, 1.2 Hz, 1H), 4.36 (ddd, *J* = 7.1, 3.6, 1.1 Hz, 1H), 4.18 (dd, *J* = 7.1, 1.1 Hz, 1H), 3.79 (s, 3H), 3.25 (dddd, *J* = 6.4, 3.6, 2.7, 1.3 Hz, 1H), 3.16 (ddd, *J* = 12.0, 10.4, 5.2 Hz, 1H), 3.11 (d, *J* = 2.7 Hz, 1H), 2.85 (dddd, *J* = 15.5, 10.4, 3.8, 0.6 Hz, 1H), 2.72 – 2.63 (m, 1H), 2.63 - 2.55 (m, 1H), 1.54 (s, 3H), 1.33 (s, 3H), 1.29 (s, 3H). **^{13}C-RMN** (100 MHz, $CDCl_3$): δ (ppm) = 174. 0, 136.1, 135.2, 129.1, 127.3, 122.0, 121.5, 119.3, 118.7, 114.0, 111.1, 109.1, 82.3, 74.1, 63.6, 58.2, 52.3, 52.1, 37.8, 25.6, 25.3, 23.8, 19.7.

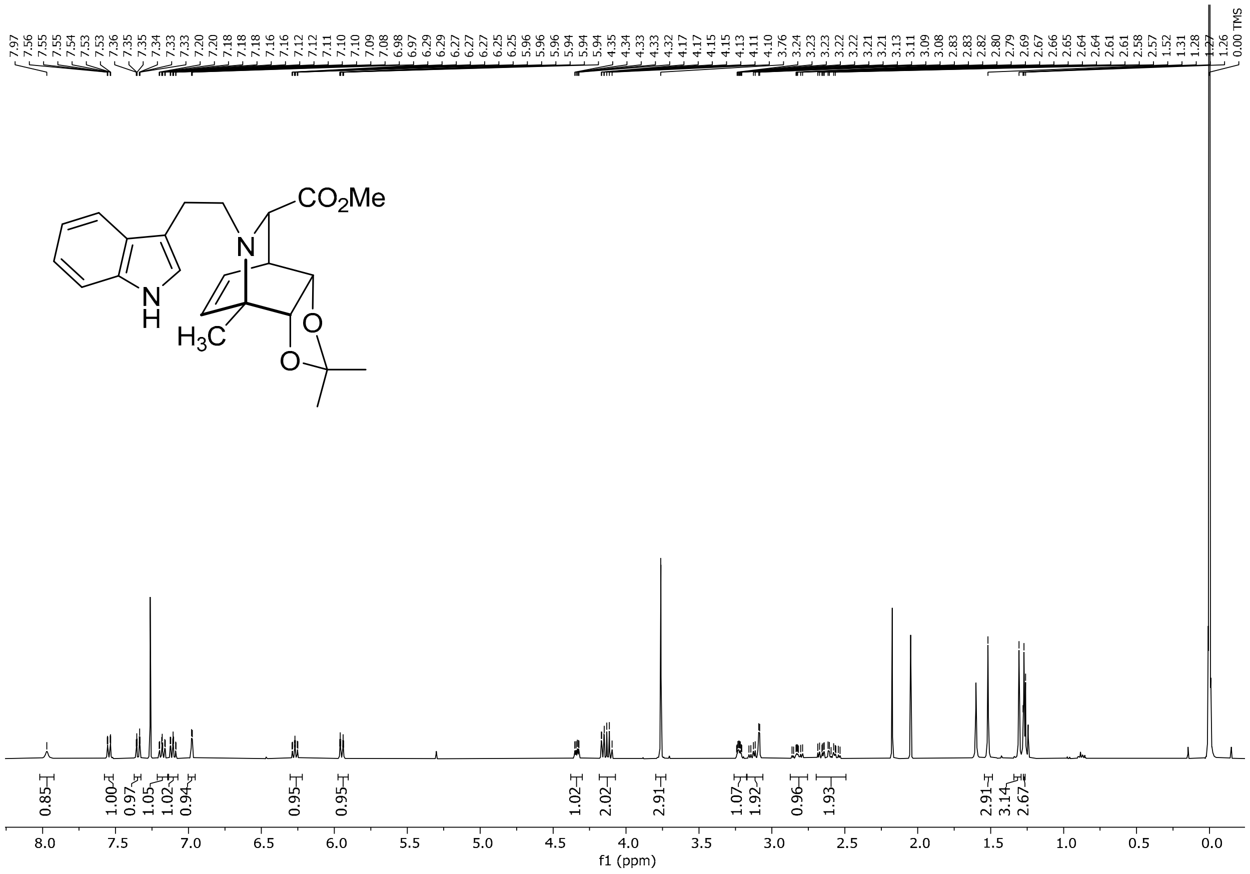

Figure S6. ^{1}H NMR of **6** in $CDCl_3$

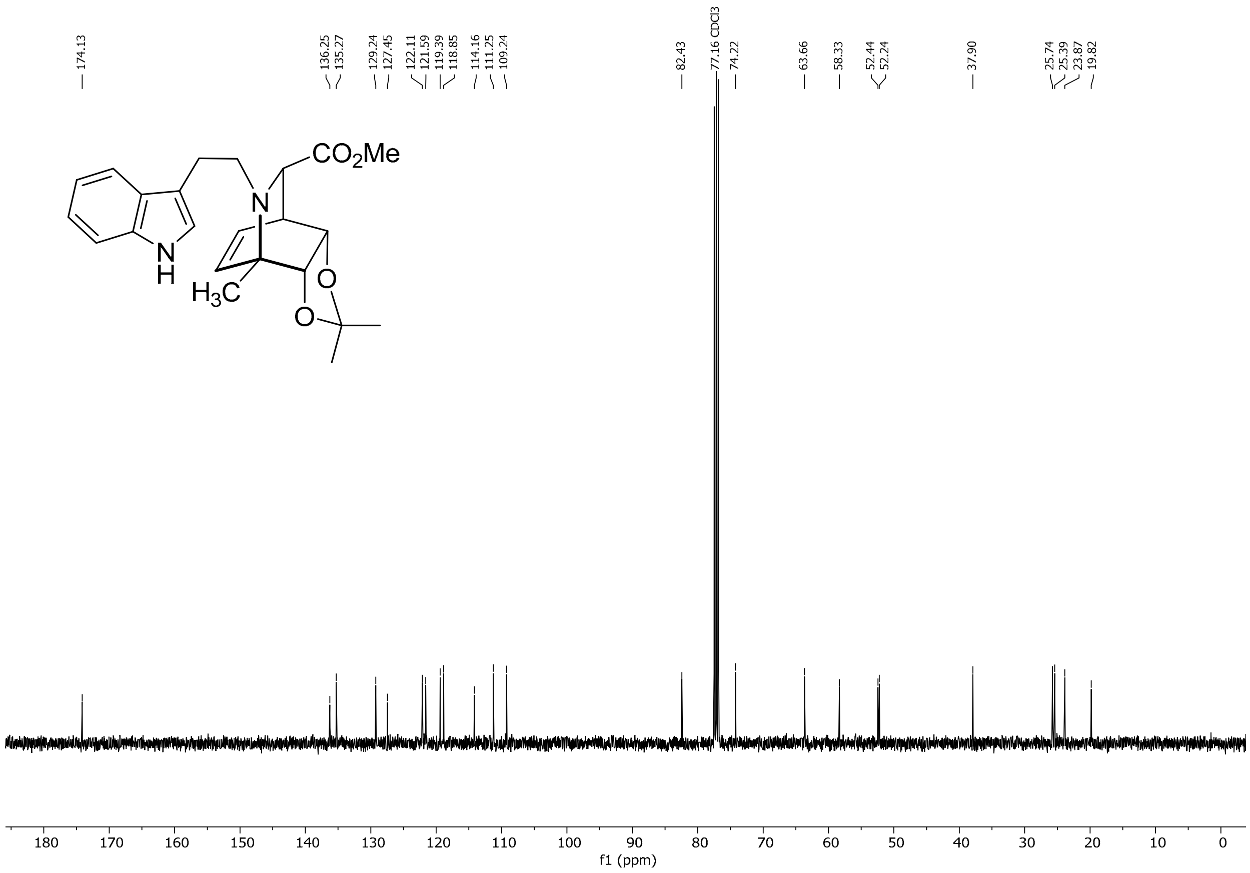

Figure S7. ^{13}C NMR of **6** in $CDCl_3$

(1*S* , 3*S*, 4*S*, 5*S*, 6*R*)Methyl-*N*-(2-(3-indolyl)ethyl)-1-methyl-5,6-isopropylidendioxy-2-azabicycle[2.2.2]-7-octenen-3-carboxylate (7)

^{1}H-RMN (400 MHz, $CDCl_3$): δ (ppm)= 8.06 (s, 1H), 7.60 (dd, J = 7.8, 1.0 Hz, 2H), 7.38 (dt, J = 8.1, 1.0 Hz, 2H), 7.21 (ddd, J = 8.2, 7.0, 1.3 Hz, 2H), 7.14 (ddd, *J* = 8.2, 7.1, 1.1 Hz, 1H), 7.04 (d, J = 2.4 Hz, 1H), 6.14 (dt, J = 8.1, 1.4 Hz, 2H), 6.02 (dd, *J* = 8.1, 1.8 Hz, 1H), 4.39 (ddd, J = 7.2, 3.5, 1.0 Hz, 2H), 4.18 (dd, J = 7.2, 1.1 Hz, 1H), 3.77-3.73 (m, 4H), 3.38 (ddd, *J* = 12.2, 6.8, 3.7 Hz, 1H); 3,30 (ddt, *J* = 5.3, 3.5, 1.9 Hz, 1H); 3,24 (d, *J* = 2.0 Hz, 1H); 2.94 – 2.85 (m, 1H), 2.85 – 2.76 (m, 1H), 2.67 (ddd, J = 12.4, 10.9, 5.1 Hz, 1H), 1.56 (s, 4H), 1.34 (s, 4H), 1.29 (s, 3H). **^{13}C-RMN** (100 MHz, $CDCl_3$): δ (ppm) = 174.4, 138.9, 136.2, 127.4, 127.1, 122.0, 121.8, 119.3, 118.7, 114.1, 111.2, 108.7, 78.4, 63.5, 56.9, 52.3, 51.6, 39.5, 31.0, 26.7, 25.6, 25.3, 20.4.

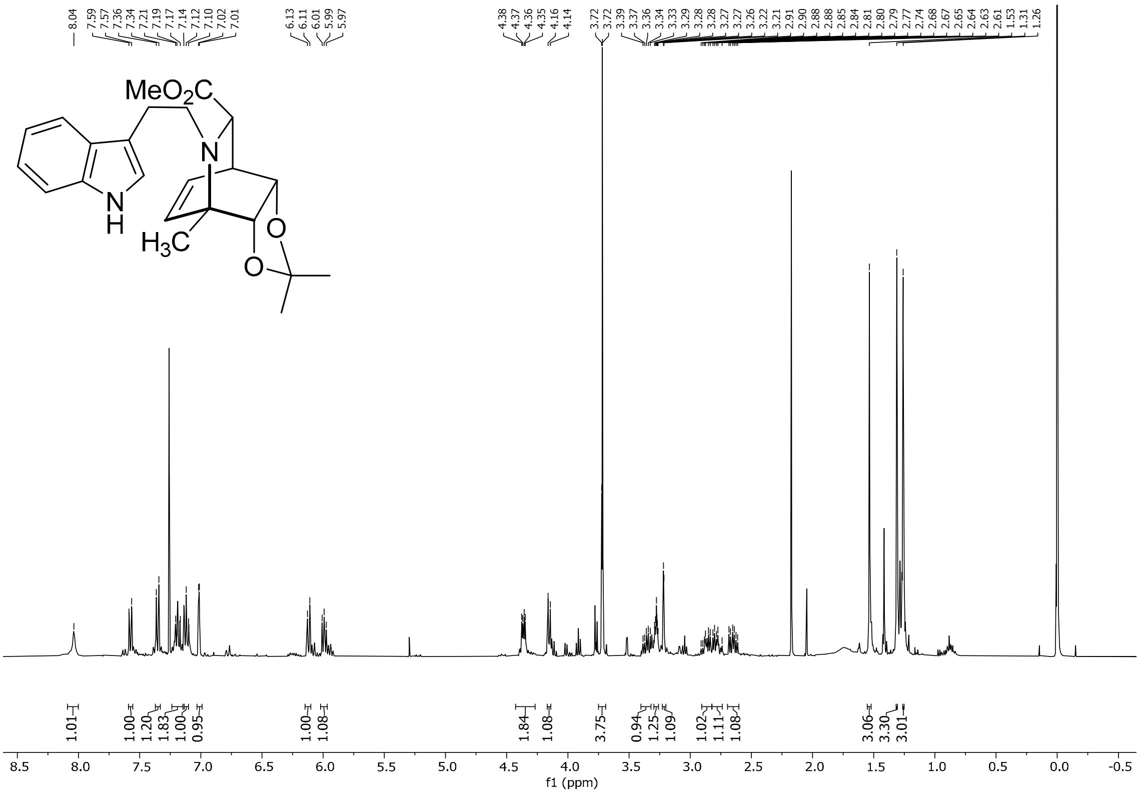

Figure S8. ^{1}H NMR of **7** in $CDCl_3$

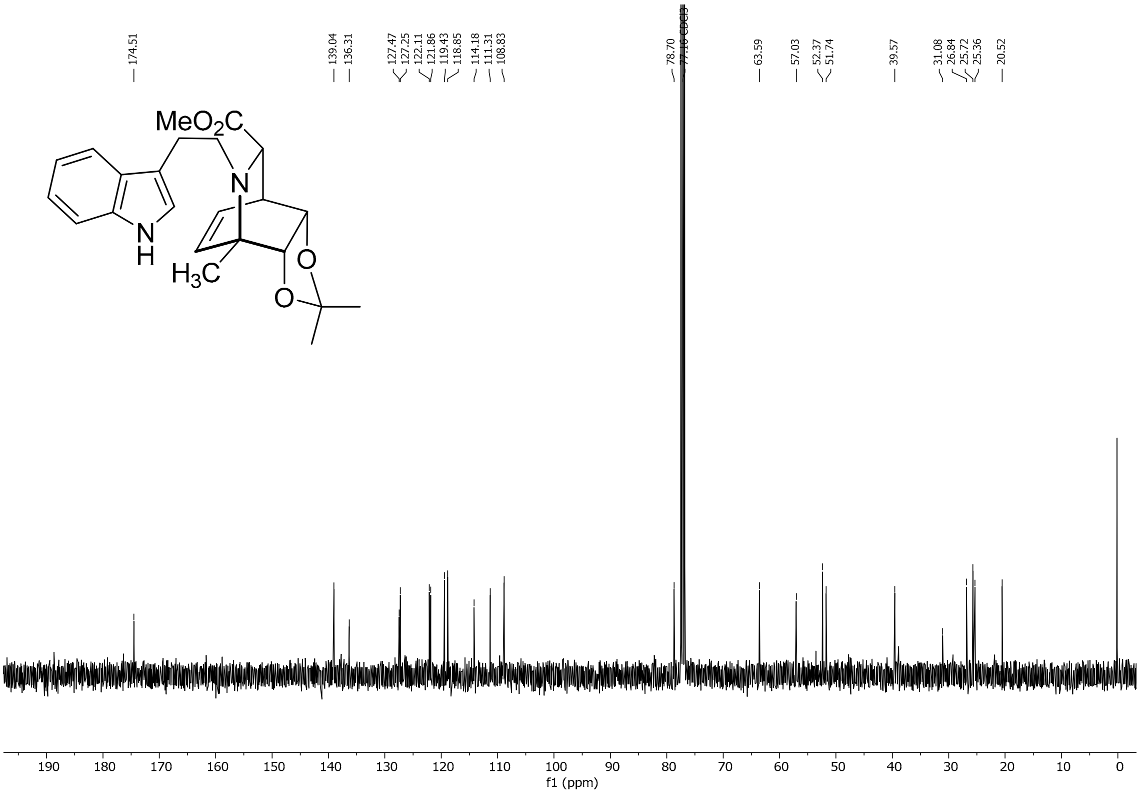

Figure S9. ^{13}C NMR of **7** in $CDCl_3$

(1*S*, 4*S*, 5*R*, 6*S*)-1-Methyl-3-hydroxymethyl-5,6-isopropylidendioxy-*N*-tosyl-2-azabicycle[2.2.2]-7-octene (8, 7:3 exo:endo)

8 *exo* (3*R*): ^{1}H-RMN (400 MHz, $CDCl_3$): δ (ppm)= 7.67 (d, *J* = 8.3 Hz, 2H), 7.28 (d, *J* = 8.3 Hz, 2H), 6.02 (dd, *J* = 8.1, 8.1 Hz, 1H), 5.56 (d, *J* = 8.1 Hz, 1H), 4.64 (dd, *J* = 7.1, 3.8 Hz, 1H), 4.10 (dd, *J* = 11.4, 5.2 Hz, 1H), 4.04 (d, *J* = 7.1 Hz, 1H), 3.83 (dd, *J* = 11.4, 6.3 Hz, 1H), 3.52 (ddd, *J* = 6.3, 5.2, 3.3 Hz, 1H), 3.27 (ddd, *J* = 3.8, 3.4, 1.2 Hz, 1H), 1.64 (s, 3H), 1.26 (s, 3H), 1.24 (s, 3H). **^{13}C-RMN** (100 MHz, $CDCl_3$): δ (ppm) = 143.7, 137.5, 134.7, 130.4, 129.5, 128.1, 127.2, 109.7, 81.3, 72.9, 65.6, 59.8, 37.2, 25.5, 25.3, 21.7, 20.3. **8 *endo* (3*S*): ^{1}H-RMN** (400 MHz, $CDCl_3$): δ (ppm) = 7.75 (d, *J* = 8.2 Hz, 2H), 7.33 – 7.29 (m, 2H), 6.22 (7, *J* = 7.1 Hz, 1H), 5.83 (d, *J* = 8.0 Hz, 1H), 4.52 (d, *J* = 7.2 Hz, 1H), 4.38 (d, *J* = 3.2 Hz, 1H), 4.03 – 4.00 (m, 1H), 3.94 (dd, *J* = 10.6, 5.0 Hz, 1H), 3.58 (dd, *J* = 8.1, 2.6 Hz, 1H), 3.40 – 3.35 (m, 1H), 2.44 (s, 3H), 1.28 (s, 3H), 1.23 (s, 3H). **^{13}C-RMN** (100 MHz, $CDCl_3$): δ (ppm) = 143.5, 140.4 135.3, 131.1, 129.7, 127.2, 109.2, 79.0, 76.2, 65.4, 59.2, 57.6, 38.6, 21.6, 21.5, 20.0.

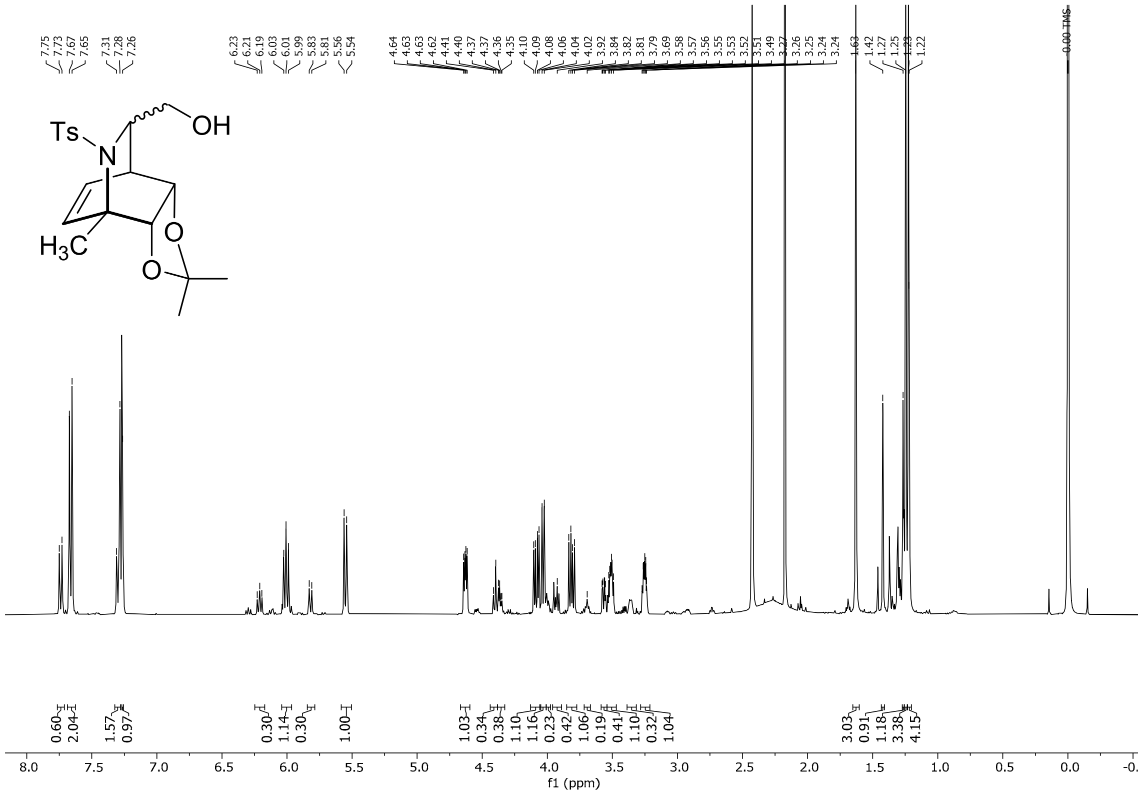

Figure S10. ^{1}H NMR of **8 (7:3 exo:endo)** in $CDCl_3$

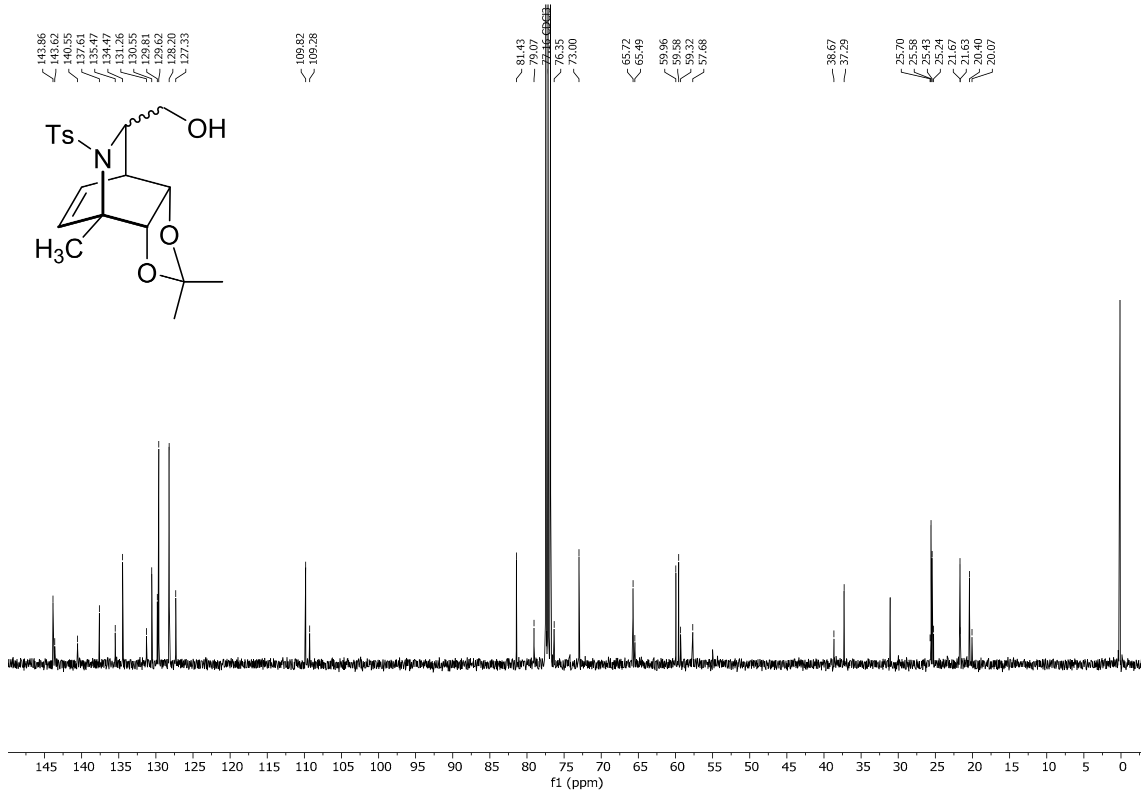

Figure S11. ^{13}C NMR of **8** (7:3 exo:endo) in $CDCl_3$

(1*S* , 4*S*, 5*R*, 6*S*)-1-Methyl-3-(dimethyl(1,1,2-trimethylpropyl)silyloxy)methyl-5,6-isopropylidendioxy-2-azabicycle[2.2.2]7-octene (9, 7:3 exo:endo, only major isomer is informed)

9exo (3S): ^{1}**H-RMN** (400 MHz, $CDCl_3$): δ (ppm)= 7.64 (d, *J* = 8.0 Hz, 2H), 7.25 (d, *J* = 7.7 Hz, 2H), 6.01 (ddd, *J* = 7.8, 6.8, 0.9 Hz, 1H), 5.53 (dt, *J* = 8.1, 1.2 Hz, 1H), 4.67 (dd, *J* = 6.8, 3.9 Hz, 1H), 4.27 (dd, *J* = 10.0, 4.9 Hz, 1H), 4.00 (dd, *J* = 7.0, 1.1 Hz, 1H), 3.53 (t, *J* = 10.0 1H), 3.46 (ddd, *J* = 10.1, 5.0, 3.1 Hz, 1H), 3.37 (dtd, *J* = 6.7, 3.5, 1.2 Hz, 1H), 1.66 (dq, *J* = 8.9, 6.9 Hz, 1H), 2.4 (s, 3H), 0.92 (s, 3H), 0.93 (s, 3H), 0.91 (s, 3H), 0.90 (s, 3H), 0.89 (s, 6H), 0.15 (s, 6H). ^{13}**C-RMN** (100 MHz, $CDCl_3$) δ (ppm): 143.4, 138.1, 134.7, 130.6, 129.4, 128.1, 109.5, 81.4, 76.7, 73.1, 64.1, 59.2, 58.4, 34.9, 34.2, 25.5, 25.3, 25.1, 21.5, 20.3, 20.3, 20.2, 20.1, 18.6, -1.5, -3.3, -3.6.

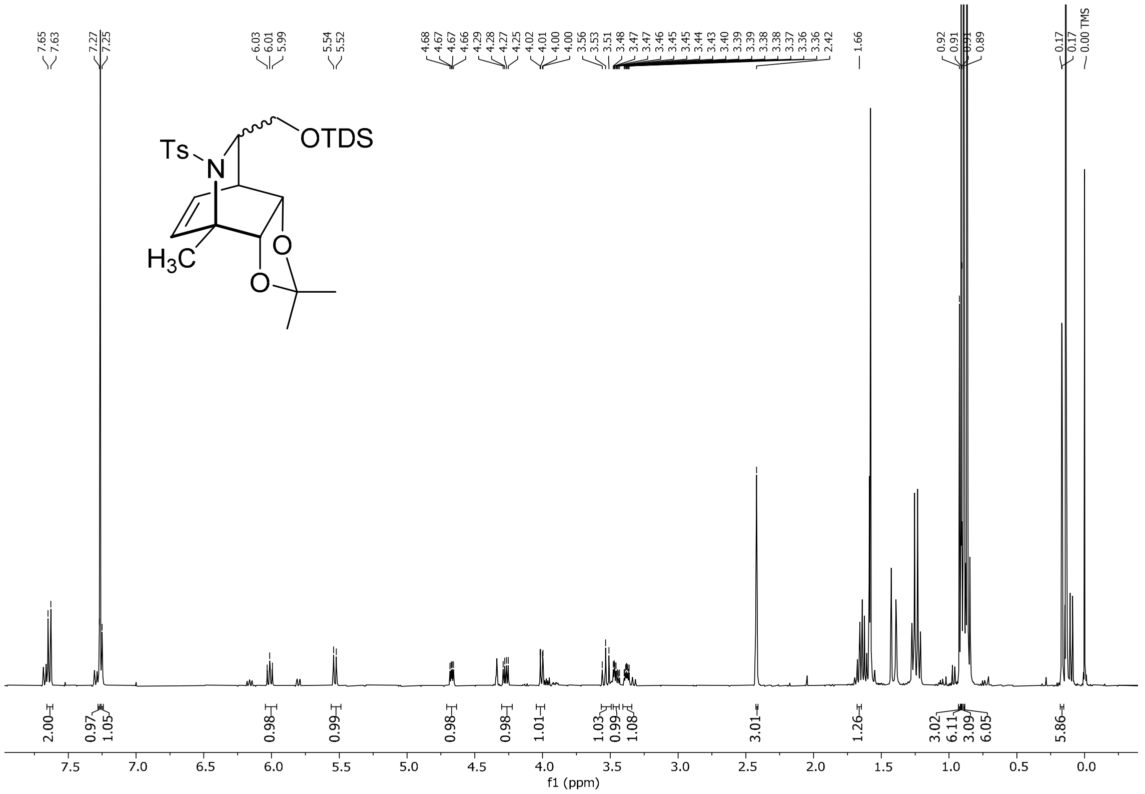

Figure S12. ^{1}H NMR of **9** (only major isomer) in $CDCl_3$

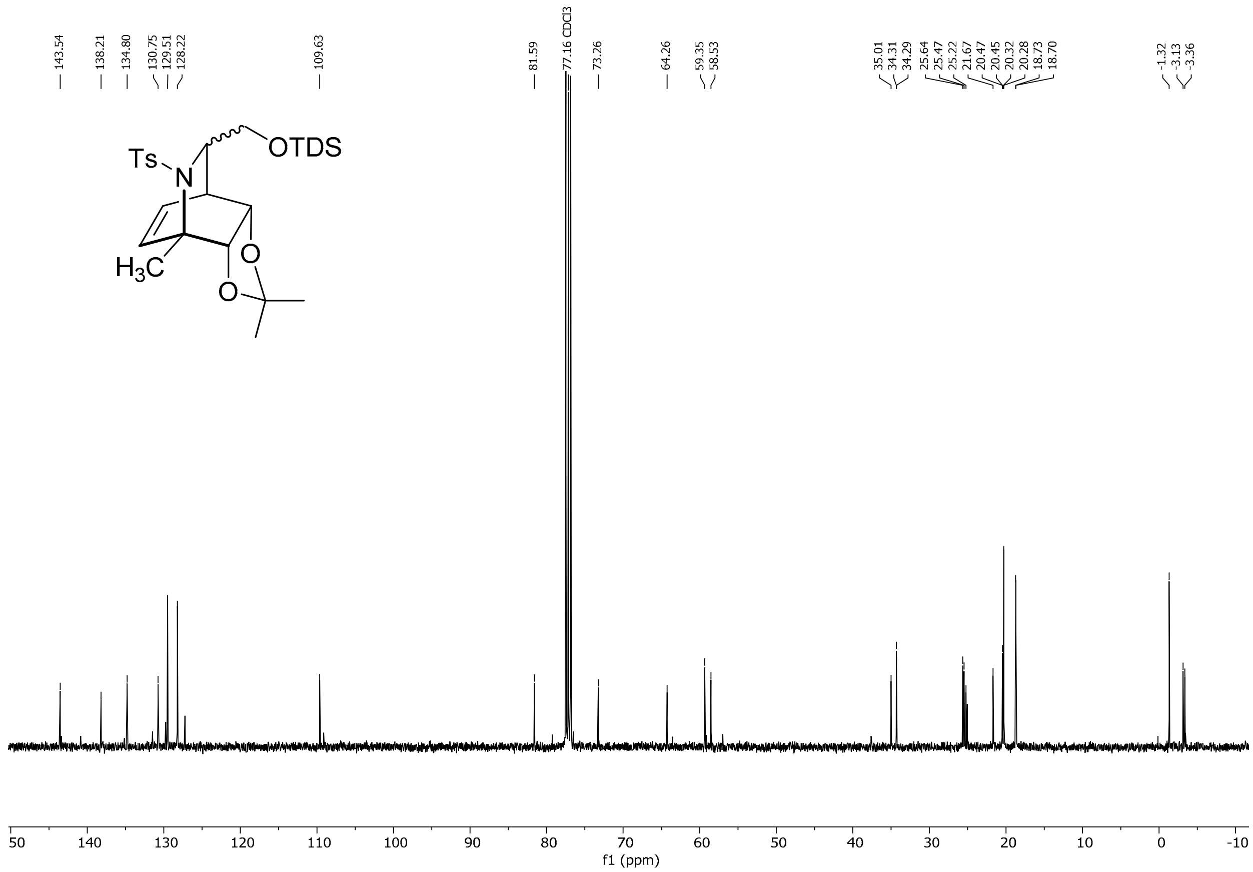

Figure S13. ^{13}C NMR of **9** (only major isomer) in $CDCl_3$

(1*S* , 3*R*, 4*S*, 5*R*, 6*S*)-1-Methyl-3-(dimethyl(1,1,2-trimethylpropyl)silyloxy)methyl-5,6-isopropylidendioxy-2-azabicycle[2.2.2]-7-octene (10 exo):

^{1}H-RMN (400 MHz, $CDCl_3$): δ (ppm) = 6.31 (ddd, *J* = 7.9, 6.7, 1.0 Hz, 1H), 5.99 (dt, *J* = 8.1, 1.2 Hz, 1H), 4.49 (ddd, *J* = 7.2, 3.5, 1.0 Hz, 1H), 3.92 (dd, J = 7.0, 1.1 Hz, 1H), 3.64 (dd, J = 10.5, 5.5 Hz, 1H), 3.45 (dd, *J* = 10.3, 9.2 Hz, 1H), 3.13 (dddd, *J* = 6.8, 3.5, 2.2, 1.2 Hz, 1H), 2.65 (ddd, J = 9.3, 5.6, 2.2 Hz, 1H), 1.64 (hept, *J* = 6.8; 1H), 1.35 (s, 3H), 1.33 (s, 3H), 1.29 (s, 3H), 0.91 (s, 3H), 0.89 (s, 3H), 0.87 (s, 6H), 0.12 (s, 3H), 0.11 (s, 3H). **^{13}C-RMN** (100 MHz, $CDCl_3$) δ (ppm) = 134.9, 132.2, 108.2, 82.6, 74.0, 64.8, 55.4, 54.0, 36.1, 34.2, 25.6, 25.2, 25.1, 21.9, 20.3, 20.3, 18.5, -3.2, -3.3.

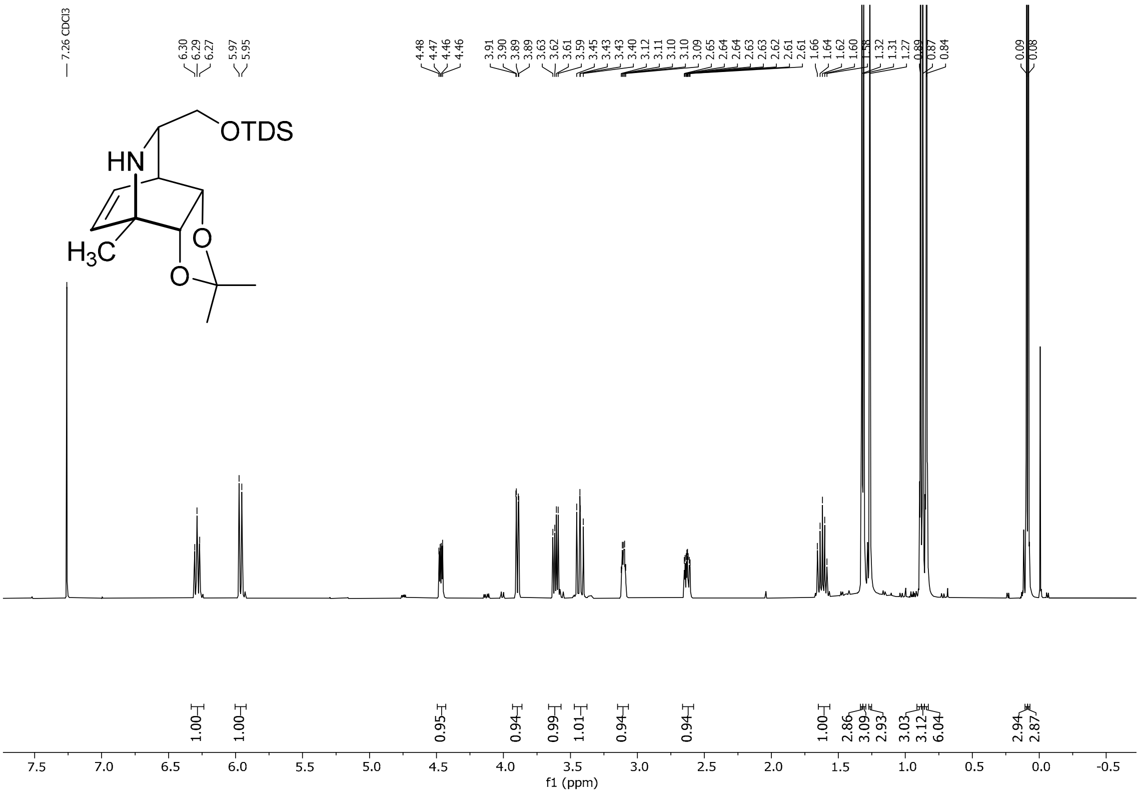

Figure S14. ^{1}H NMR of **10 exo** in $CDCl_3$

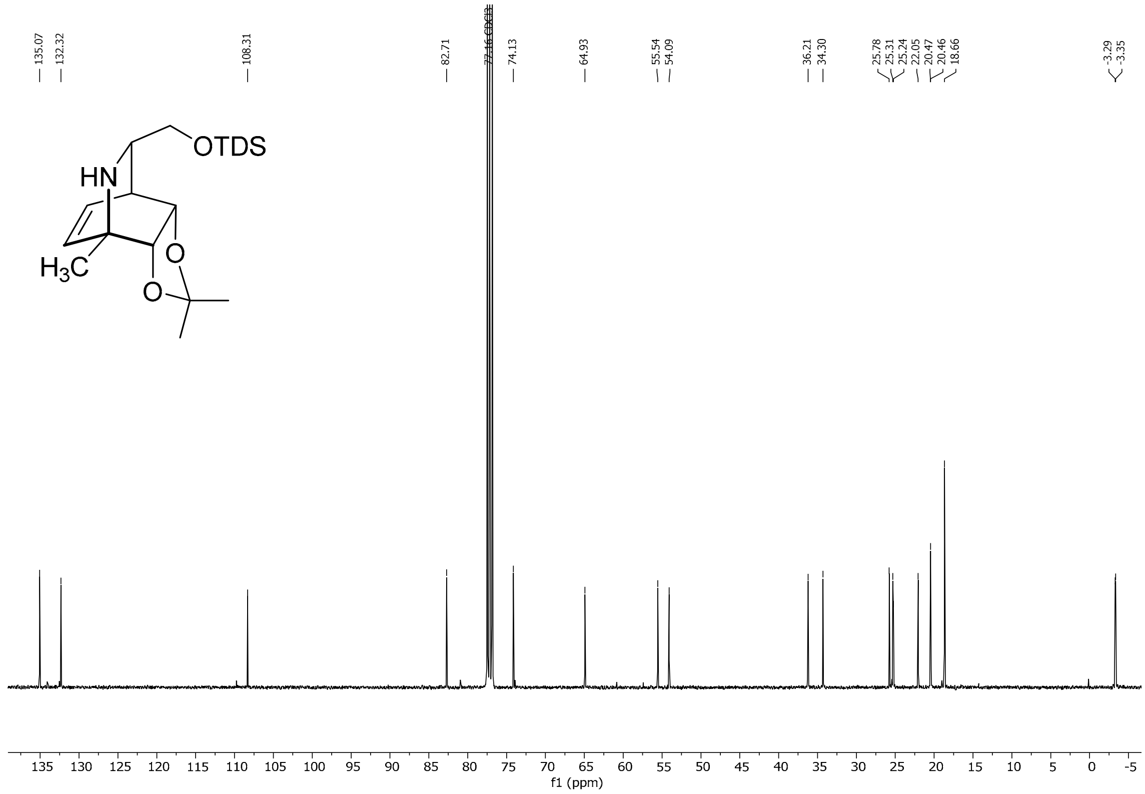

Figure S15. ^{13}C NMR of **10 exo** in $CDCl_3$

(1*S* , 3*S*, 4*S*, 5*S*, 6*R*)-1-Methyl-3-(dimethyl(1,1,2-trimethylpropyl)silyloxy)methyl-5,6-isopropylidendioxy-2-azabicycle[2.2.2]-7-octene (10 endo):

^{1}H-RMN (400 MHz, $CDCl_3$): δ (ppm)= 5.96 – 6.04 (m, 2H), 4.32 (ddd, *J* = 7.1, 3.5, 0.9 Hz, 1H), 3.95 (dd, *J* = 7.1, 1.0 Hz, 1H), 3.28 (dd, *J* = 9.5, 6.1 Hz, 1H), 3.19 (dd, *J* = 9.5, 8.2 Hz, 1H), 3.08 (ddt, *J* = 5.4, 3.5, 1.8 Hz, 1H), 2.92 (dd, *J* = 8.2, 6.2 Hz, 1H), 1.59 (q, *J* = 6.8, 6.8 Hz, 1H), 1.31 (s, 6H), 1.28 (s, 3H), 0.86 (s, 3H), 0.85 (s, 3H), 0.81 (s, 6H), 0.04 (s, 6H). **^{13}C-RMN** (100 MHz, $CDCl_3$) δ (ppm) = 136.7, 128.6, 109.3, 83.4, 78.5, 65.3, 54.8, 53.8, 37.8, 34.6, 31.4, 26.1, 25.6, 25.6, 22.4, 20.8, 20.7, 19.0, 19.0.

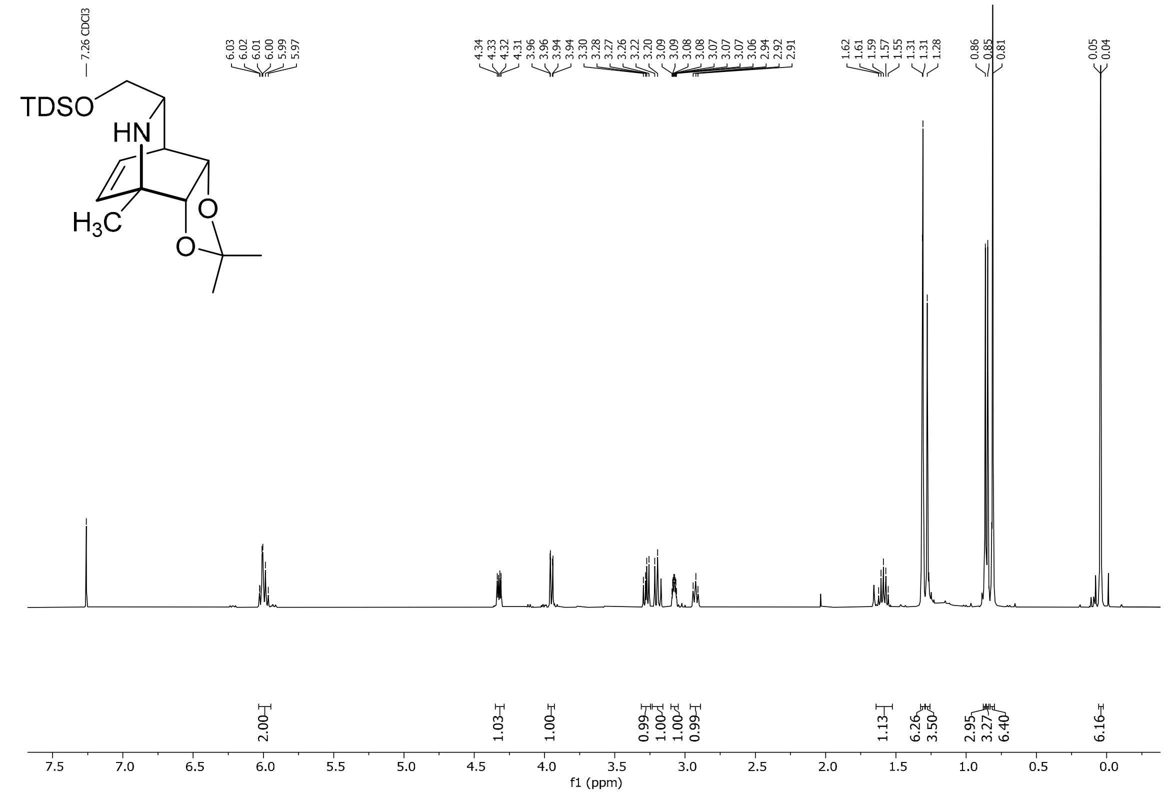

Figure S16. ^{1}H NMR of **10 endo** in $CDCl_3$

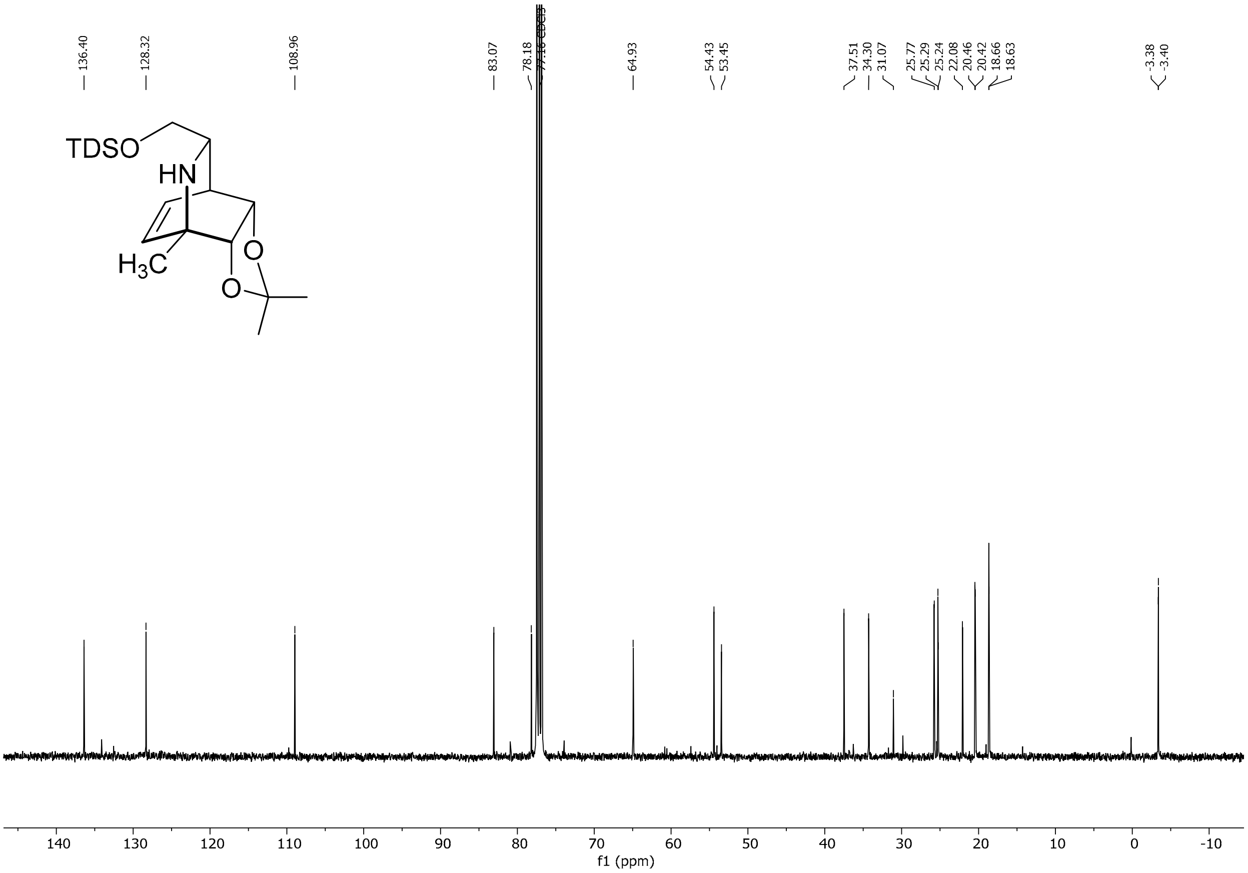

Figure S17. ^{13}C NMR of **10 endo** in $CDCl_3$

(1*S* , 3*R,* 4*S,* 5*S*, 6*R*)-*N*-(2-(3-indolyl)ethyl)-1-methyl-3-(dimethyl(1,1,2-trimethylpropyl)silyloxy)methyl-5,6-isopropylidendioxy-2-azabicycle[2.2.2]-7-octene (11)

^{1}H-RMN (400 MHz, $CDCl_3$): δ (ppm)= 7.97 (s, 1H); 7.55 (d, *J* = 7.8 Hz, 1H), 7.35 (dt, *J* = 8.1, 0.9 Hz, 1H), 7.18 (ddd, *J* = 8.2, 7.1, 1.2 Hz, 1H), 7.11 (ddd, *J* = 8.0, 7.0, 1.1 Hz, 1H), 6.95 (d, *J* = 2.3 Hz, 1H), 6.27 (ddd, *J* = 7.8, 6.7, 0.9 Hz, 1H), 5.95 (dt, *J* = 8.0, 1.1 Hz, 1H), 4.53 (dd, *J* = 7.2, 3.5 Hz, 1H), 3.92 (dd, *J* = 7.2, 1.0 Hz, 1H), 3.65 (dd, *J* = 10.5, 5.1 Hz, 1H), 3.41 (t, *J* = 10.3 Hz, 1H), 3.20 (dddd, *J* = 6.6, 3.7, 2.7, 1.2 Hz, 1H), 3.01 (ddd, *J* = 13.3, 11.7, 4.2 Hz, 1H), 2.87 (ddd, *J* = 13.1, 11.2, 4.3 Hz, 1H), 2.74 (ddd, *J* = 13.3, 11.1, 5.0 Hz, 1H), 2.60 (ddd, *J* = 13.3, 11.1, 5.0 Hz, 1H), 2.46 (ddd, *J* = 10.2, 5.1, 2.6 Hz, 1H), 1,58-1.69 (m, 3H), 1.48 (s, 3H), 1.33 (s, 3H), 1.29 (s, 3H), 0.91 (s, 3H), 0,89 (s, 3H), 0,86 (s, 6H), 0,10 (s, 3H). **^{13}C-NMR** (100 MHz, $CDCl_3$): δ (ppm) = 136.2, 134.5, 130.6, 127.4, 121.0, 121.3, 119.3, 118.7, 114.6, 111.1, 108.7, 82.8, 73.8, 63.9, 61.6, 58.1, 51.9, 34.9, 34.2, 30.9, 25.7, 25.3, 25.1, 24.8, 20.4, 19.8, 18.6, -3.2, -3.3.

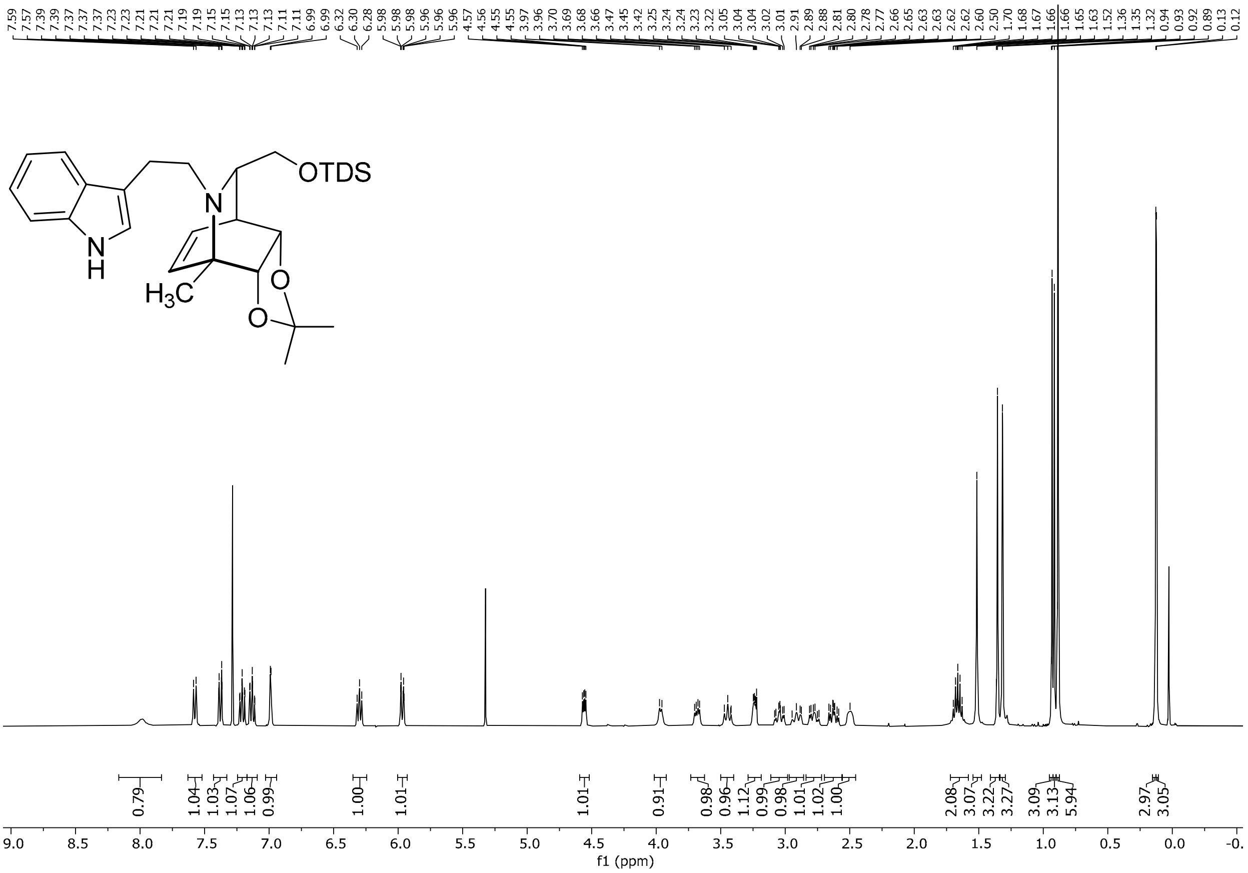

Figure S18. ^{1}H NMR of **11** in $CDCl_3$

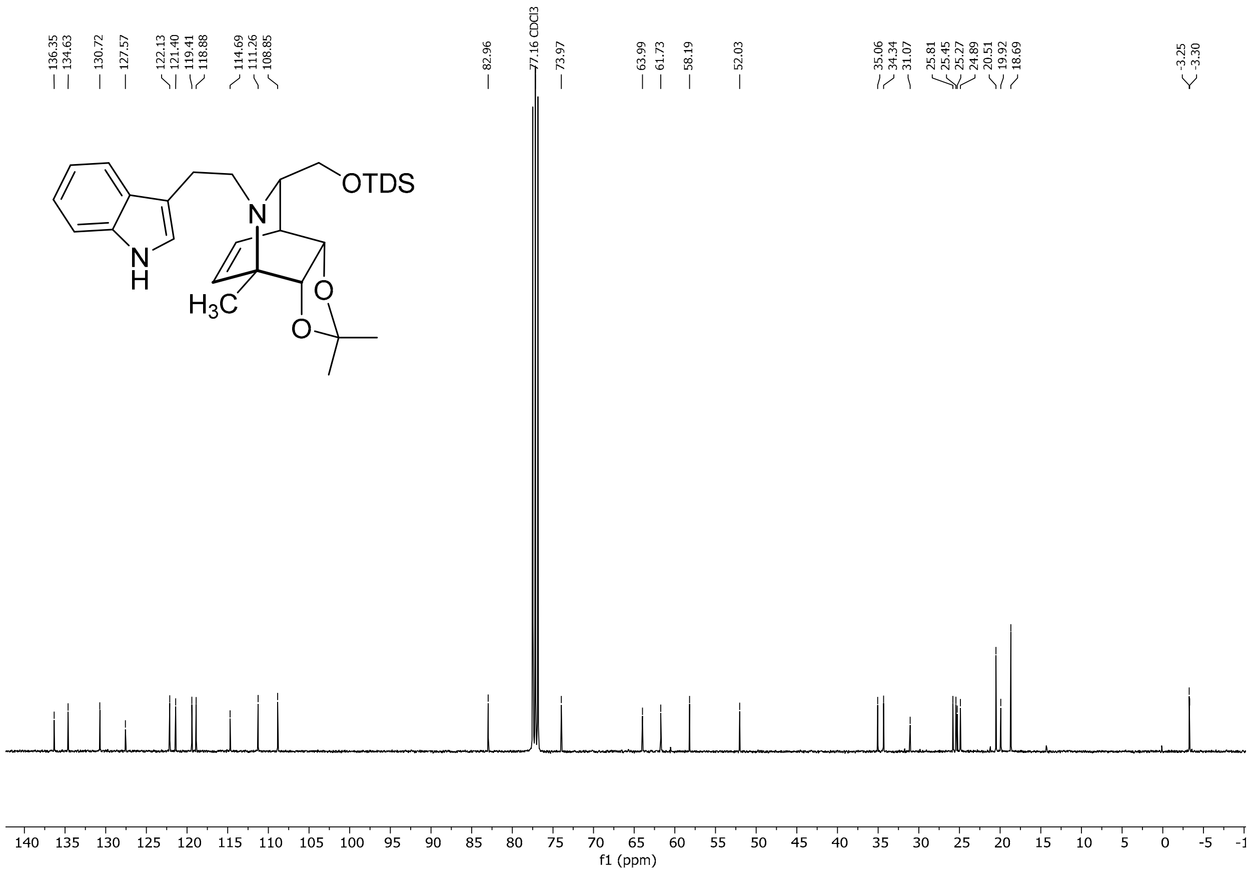

Figure S19. ^{13}C NMR of **11** in $CDCl_3$

(1*S* , 3*S,* 4*S*, 5*S*, 6*R*)-*N*-(2-(3-indolyl)ethyl)-1-methyl-3-(dimethyl(1,1,2-trimethylpropyl)silyloxy)methyl-5,6-isopropylidendioxy-2-azabicycle[2.2.2]-7-octene (12)

^{1}H-RMN (400 MHz, $CDCl_3$): δ (ppm)= 7.98 (s, 1H), 7.55 (d, *J* = 7.9 Hz, 1H), 7.37 (dt, *J* = 8.1, 0.9 Hz, 1H), 7.20 (ddd, *J* = 8.2, 7.0, 1.2, Hz, 1H), 7.13 (ddd, *J* = 8.0, 7.0, 1.0 Hz, 1H), 7.01 (d, *J* = 2.3 Hz, 1H), 6.06 (dd, *J* = 8.1, 6.1, Hz, 1H), 6.00 (ddd, *J* = 8.1, 1.8, 1.0 Hz, 1H), 4.32 (ddd, *J* = 7.2, 3.5, 0.7 Hz, 1H), 4.23 (dd, *J* = 7.2, 1.0 Hz, 1H), 3.51 (dd, *J* = 9.8, 4.2 Hz, 1H), 3.33 – 3.23 (m, 3H), 2.98 – 2.80 (m, 2H), 2.61 (ddd, *J* = 12.5, 10.6, 6.0 Hz, 1H), 2.56 – 2.50 (m, 1H), 1.68 – 1.55 (m, 3H), 1.49 (s, 3H), 1.33 (s, 3H), 1.26 (s, 3H), 0.90 (s, 3H), 0.88 (s, 3H), 0.85 (s, 3H), 0.84 (s, 3H), 0.08 (s, 3H), 0.07 (s, 3H). **^{13}C-RMN** (100 MHz, $CDCl_3$): δ (ppm) = 137.8, 136.2, 129.6, 127.4, 122.1, 121.5, 119.4, 118.7, 114.3, 111.2, 108.1, 78.6, 65.6, 63.5, 57.0, 51.9, 37.5, 34.2, 28.3, 25.6, 25.2, 25.1, 20.7, 20.4, 20.4, 18.6, -3.3, -3,3.

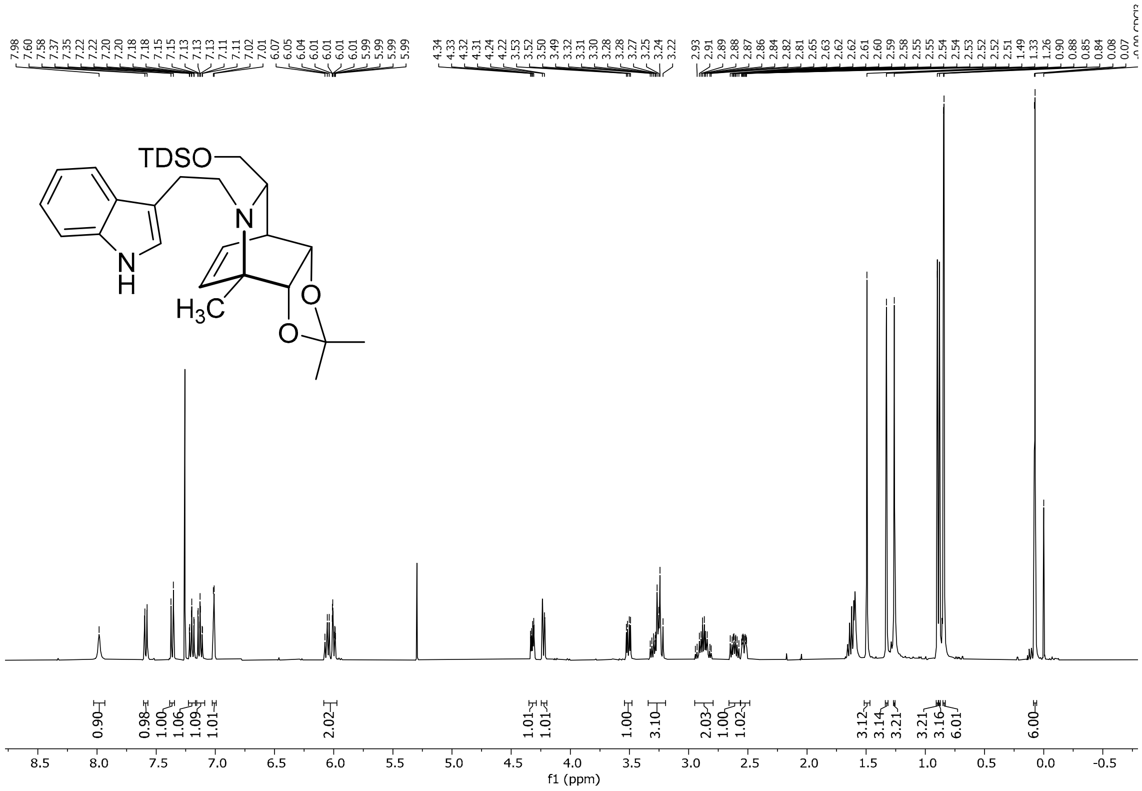

Figure S20. ^{1}H NMR of **12** in $CDCl_3$

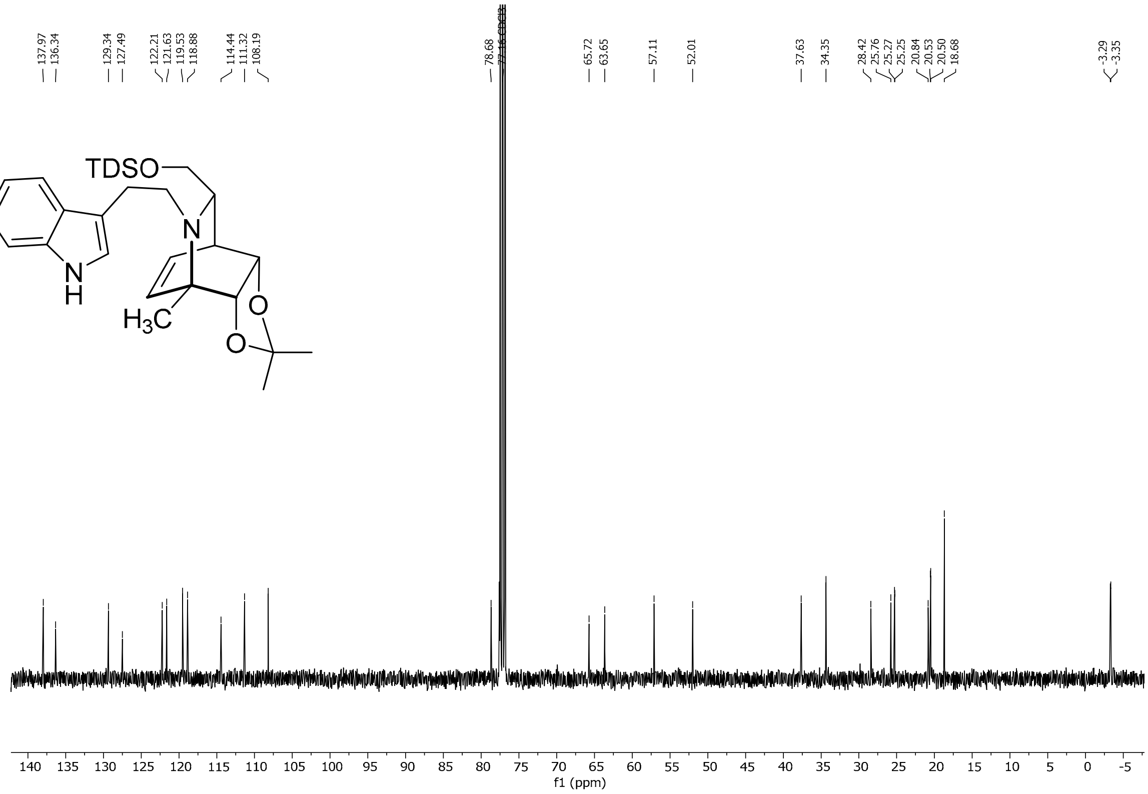

Figure S21. ^{13}C NMR of **12** in $CDCl_3$

(1*S* , 3*R,* 4*S*, 5*S*, 6*R*)-*N*-(2-(3-indolyl)ethyl)-1-methyl-3-hydroxymethyl-5,6-isopropylidendioxy-2-azabicycle[2.2.2]-7-octene (13)

^{1}H-RMN (400 MHz, $CDCl_3$): δ (ppm)= 8.03 (s, 1H), 7.53 (dq, *J* = 7.8, 0.9 Hz, 1H), 7.37 (dt, *J* = 8.1, 1.0 Hz, 1H), 7.22 (ddd, *J* = 8.2, 7.0, 1.2 Hz, 1H), 7.13 (ddd, *J* = 8.0, 7.0, 1.1 Hz, 1H), 6.99 (d, *J* = 2.4 Hz,1H), 6.30 (ddd, *J* = 7.9, 6.5, 1.0 Hz, 1H), 5.99 (dt; *J* = 7.9, 1.0 Hz, 1H), 4.72 (ddd, *J* = 7.1, 3.8, 0.9 Hz, 1H), 3.93 (dd, *J* = 7.1, 1.0 Hz, 1H), 3.64 (dd, *J* = 10.9, 6.3 Hz, 1H), 3.56 (dd, *J* = 10.8, 2.2 Hz, 1H), 3.15 – 3.06 (m, 1H), 2.95 (dddd, *J* = 6.5, 3.8, 2.7, 1.2 Hz, 1H), 2.93 – 2.86 (m, 1H), 2.81 – 2.66 (m, 2H), 2.55 (dt, *J* = 6.3, 2.5 Hz, 1H), 1.52 (s, 3H), 1.31 (s, 3H), 1.27 (s, 3H). **^{13}C-RMN** (101 MHz, $CDCl_3$) δ (ppm) = 136.3, 134.1, 130.8, 127.3, 122.1, 121.4, 119.3, 118.6, 114.1, 111.2, 108.9, 83.1, 73.9, 62.0, 60.8, 58.6, 51.5, 39.7, 25.6, 25.3, 24.8, 20.0

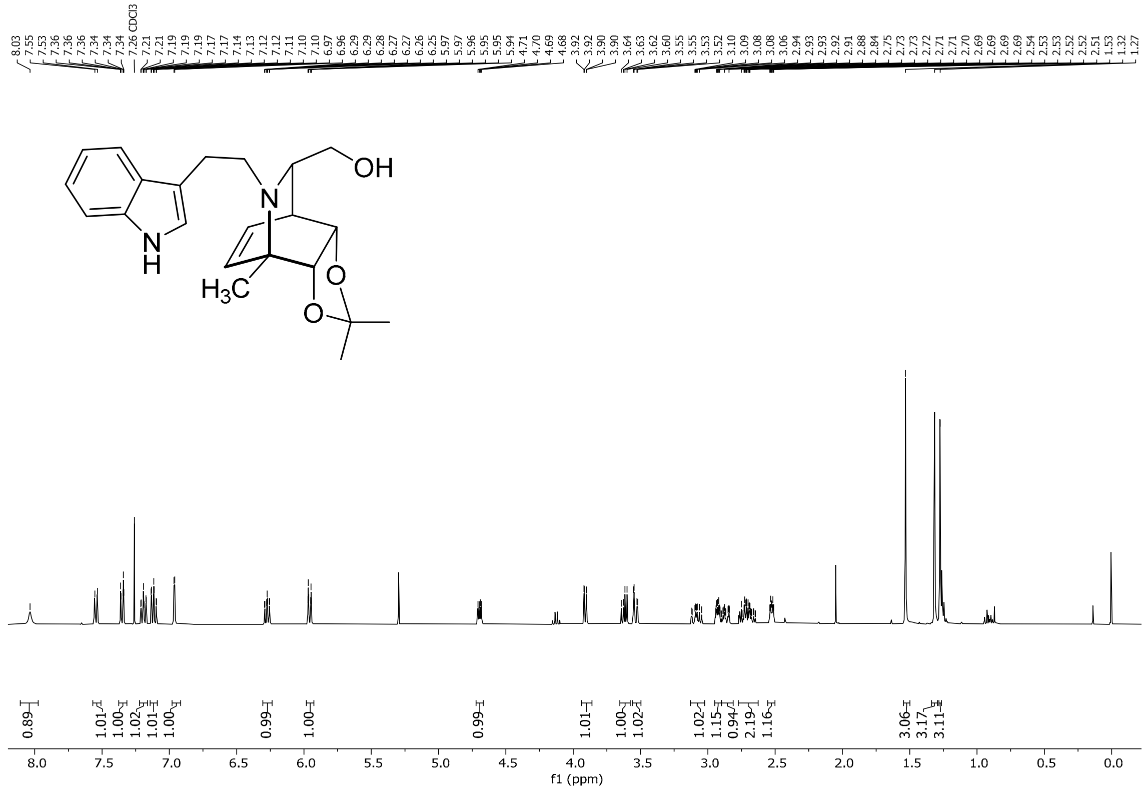

Figure S22. ^{1}H NMR of **13** in $CDCl_3$

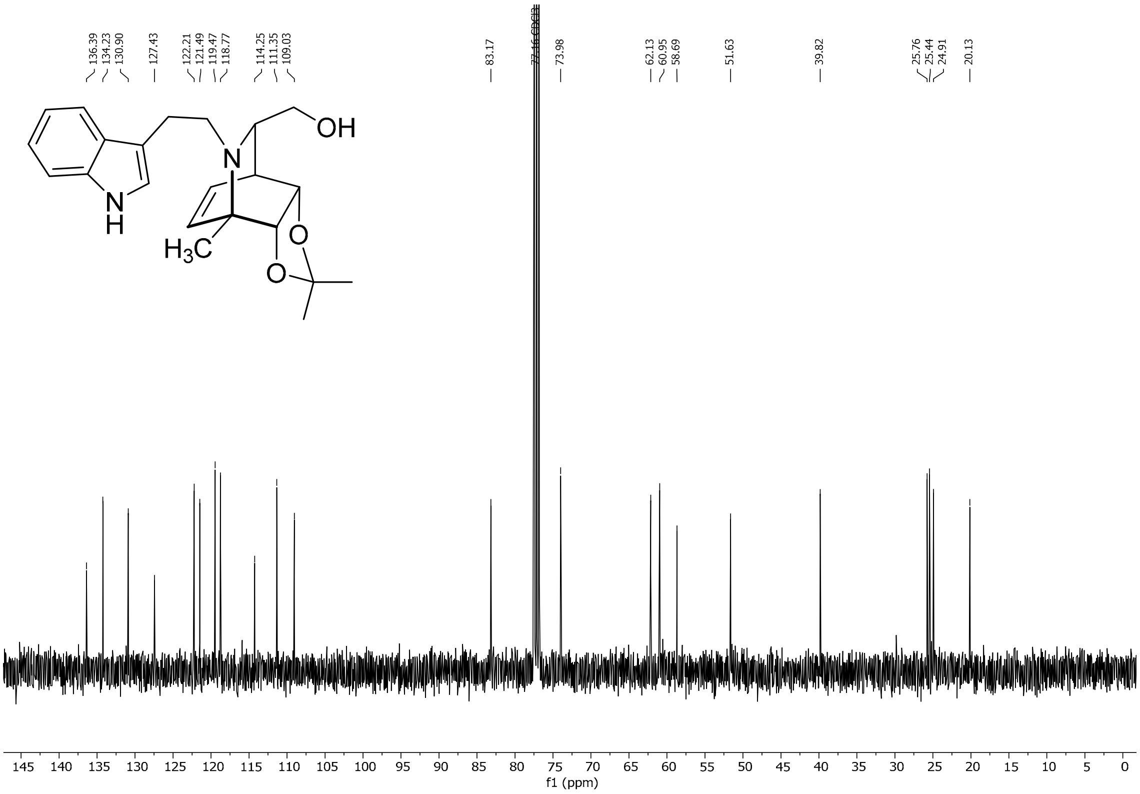

Figure S23. ^{13}C NMR of **13** in $CDCl_3$

(1*S* , 3*R,* 4*S*, 5*S*, 6*R*)-*N*-(2-(3-indolyl)ethyl)-1-methyl-3-benzyloximethyl-5,6-isopropylidendioxy-2-azabicycle[2.2.2]-7-octene (14)

^{1}H-RMN (400 MHz, $CDCl_3$): δ (ppm)= 7.97 (s, 1H), 7.57 (d, *J* = 7.8), 7.29 – 7.39 (m, 6H), 7.21 (ddd, *J* = 8.2, 7.0, 1.2 Hz, 1H), 7.13 (ddd, *J* = 8.1, 7.1, 1.1 Hz; 1H), 6.95 (d, *J* = 2.3), 6.28 (ddd, *J* = 8.0, 6.8, 1.1 Hz, 1H), 5.97 (dt, *J* = 8.0, 1.1 Hz, 1H), 4.61 (d, *J* = 11.9 Hz, 1H), 4.53 (d, *J* = 11.9 Hz, 1H), 4.45 (dd, *J* = 7.2, 3.6 Hz, 1H), 3.93 (dd, *J* = 7.2, 1.0 Hz, 1H), 3.58 (dd, *J* = 9.6, 5.3 Hz, 1H), 3.39 (t, *J* = 9.6 Hz, 1H), 3.23 (ddq, *J* = 4.8, 3.7, 1.3 Hz, 2H), 3,07 (ddd, *J* = 13.3, 12.0, 4.1 Hz, 1H), 2.88 (ddd, *J* = 13.3, 11.2, 4.1 Hz, 1H), 2.78 (ddd, *J* = 13.4, 11.2, 4.7 Hz, 1H), 2.68 (ddd, *J* = 9.5, 5.1, 2.1 Hz, 1H), 1.51 (s, 3H), 1.35 (s, 3H), 1.28 (s, 3H). **^{13}C NMR** (100 MHz, $CDCl_3$): δ (ppm) = 138.2, 136.2, 134.5, 130.4, 128.4, 127.8, 127.7, 127.4, 122.0, 121.3, 119.3, 118.8, 114.5, 111.1, 108.7, 82.7, 73.8, 73.3, 71.6, 59.3, 58.0, 51.8, 35.6, 29.7, 25.7, 25.3, 24.7, 19.8.

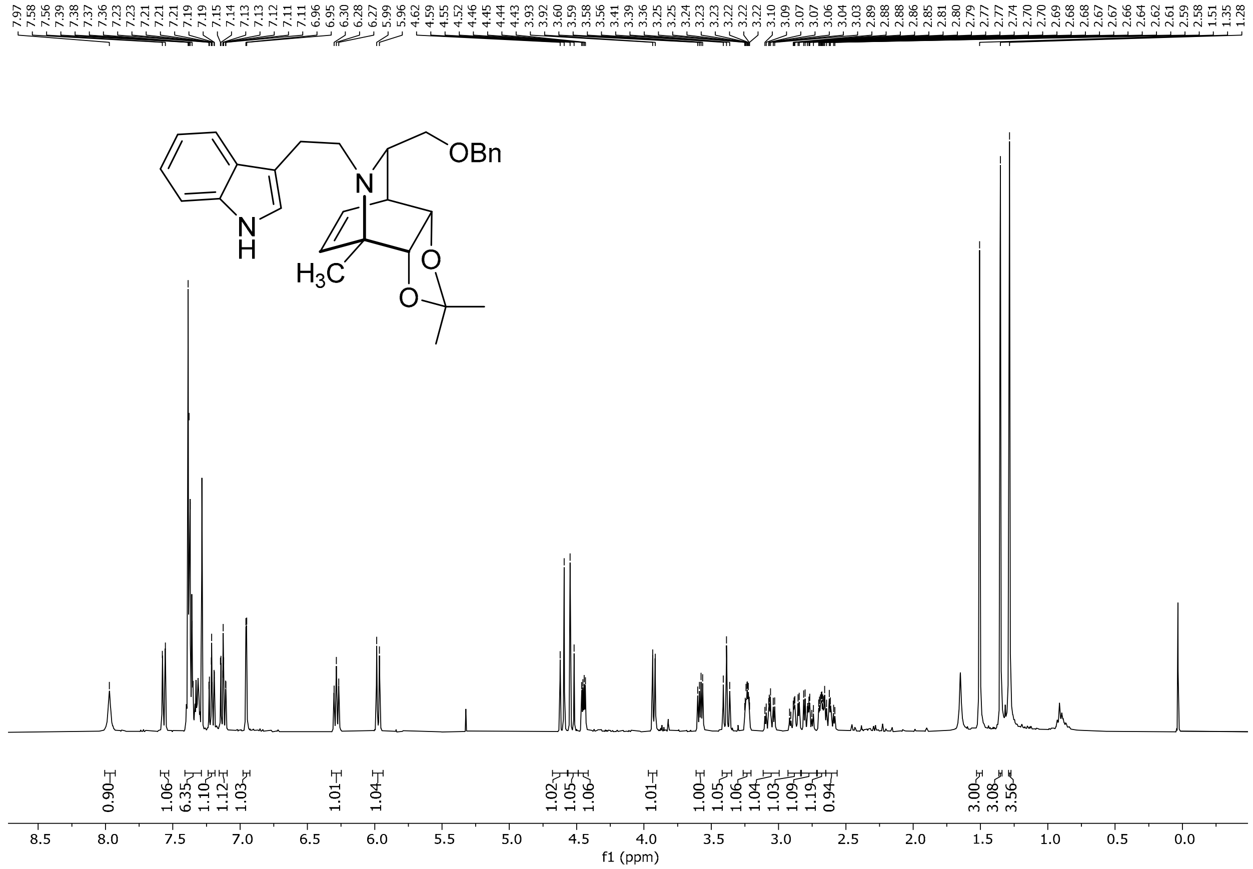

Figure S24. ^{1}H NMR of **14** in $CDCl_3$

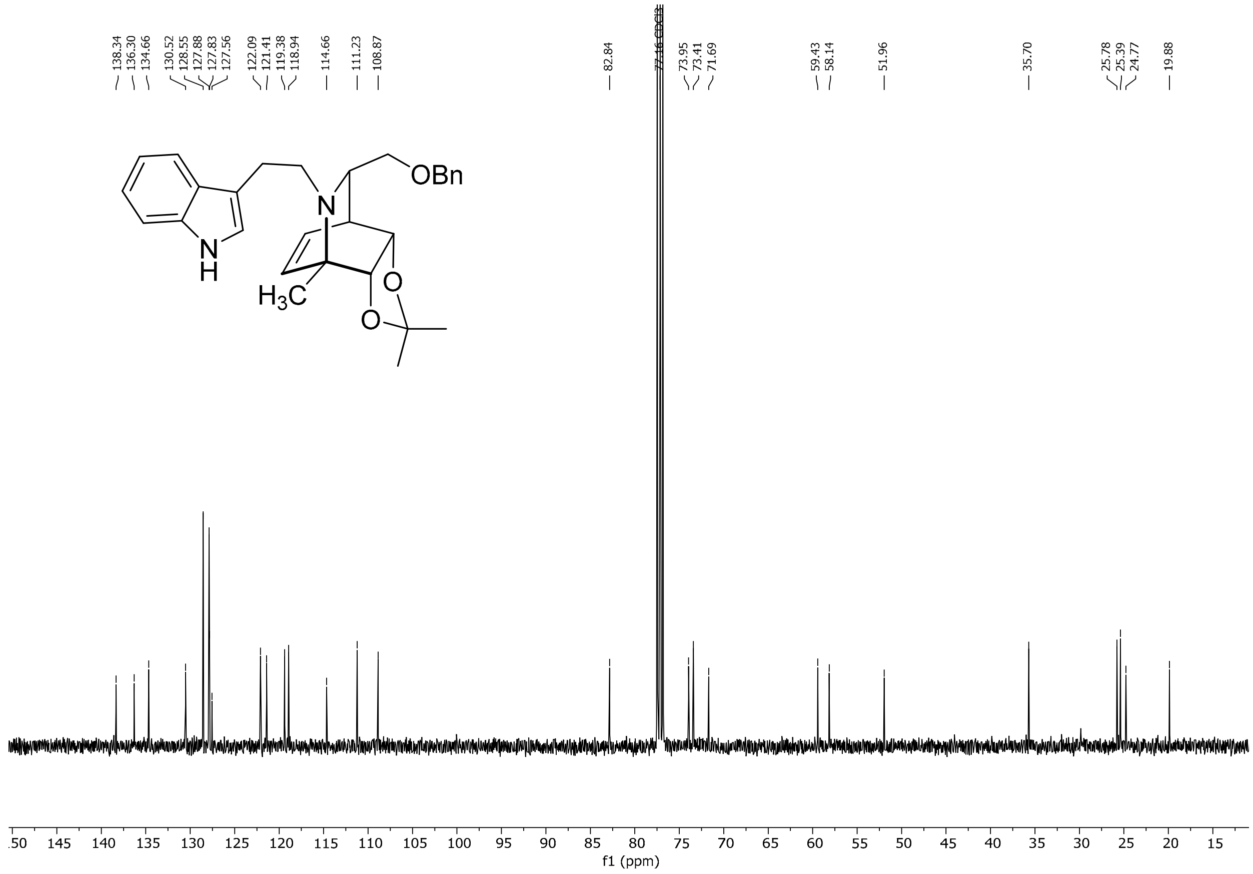

Figure S25. ^{1}H NMR of **14** in $CDCl_3$

(1*S* , 3*R,* 4*S*, 5*S*, 6*R*)-*N*-(2-(3-indolyl)ethyl)-1-methyl-3-(2-(1*H*-3-indolyl)acetyloxy)methyl-5,6-isopropylidendioxy-2-azabicycle[2.2.2]-7-octene (15)

^{1}H-RMN (400 MHz, $CDCl_3$): δ (ppm) = 8.07 (s, 1H), 7.98 (s, 1H), 7,64 (ddd, *J* = 7.9, 2.1, 0.9 Hz, 1H), 7.59 (ddd, *J* = 7.9, 1.8, 0.9 Hz, 1H), 7.36 (dt, *J* = 6.0, 1.1 Hz, 1H), 7.34 (dt, *J* = 5.6, 1.1 Hz, 1H), 7.24 – 7.28 (m, 2H), 7.18-7.13 (m, 2H), 7.12-7.11 (m, 1H), 6.93 (d, *J* = 2.2, 1H); 6,20 (ddd, *J* = 7.8, 6.6, 0.9 Hz; 1H), 5.95 (dt, *J* = 8.1, 1.2 Hz, 1H), 4.52 (dd, *J* = 7.2, 3.5 Hz, 1H), 4.27 (dd, *J* = 11.4, 5.6 Hz, 1H), 4.02 (dd, *J* = 11.4, 9.2 Hz, 1H), 3.94 (d, *J* = 7.1 Hz, 1H), 3.06 (ddd, *J* = 13.4, 11.9, 4.1 Hz, 1H), 2.97 (dddd, *J* = 6.5, 3.7, 2.6, 1.2 Hz, 1H), 2.91 – 2.82 (m, 1H); 2.77 (ddd, *J* = 13.5, 11.2, 4.6 Hz, 1H), 2.70 – 2,60 (m, 2H), 1,50 (s, 3H), 1.31 (s, 3H), 1.26 (s, 3H). **^{13}C-RMN** (100 MHz, $CDCl_3$) δ (ppm) = 171.9, 136.2, 136.1, 134.9, 129.9, 127.4, 127.2, 123.1, 122.2, 122.0, 121.4, 119.7, 119.3, 118.8, 118.8, 114.4., 111.2, 111.1, 108.2, 82.6, 73.7, 65.6, 58.2, 58.1, 51.1, 35.7, 31.4, 25.7, 25.3, 24.5, 19.7.

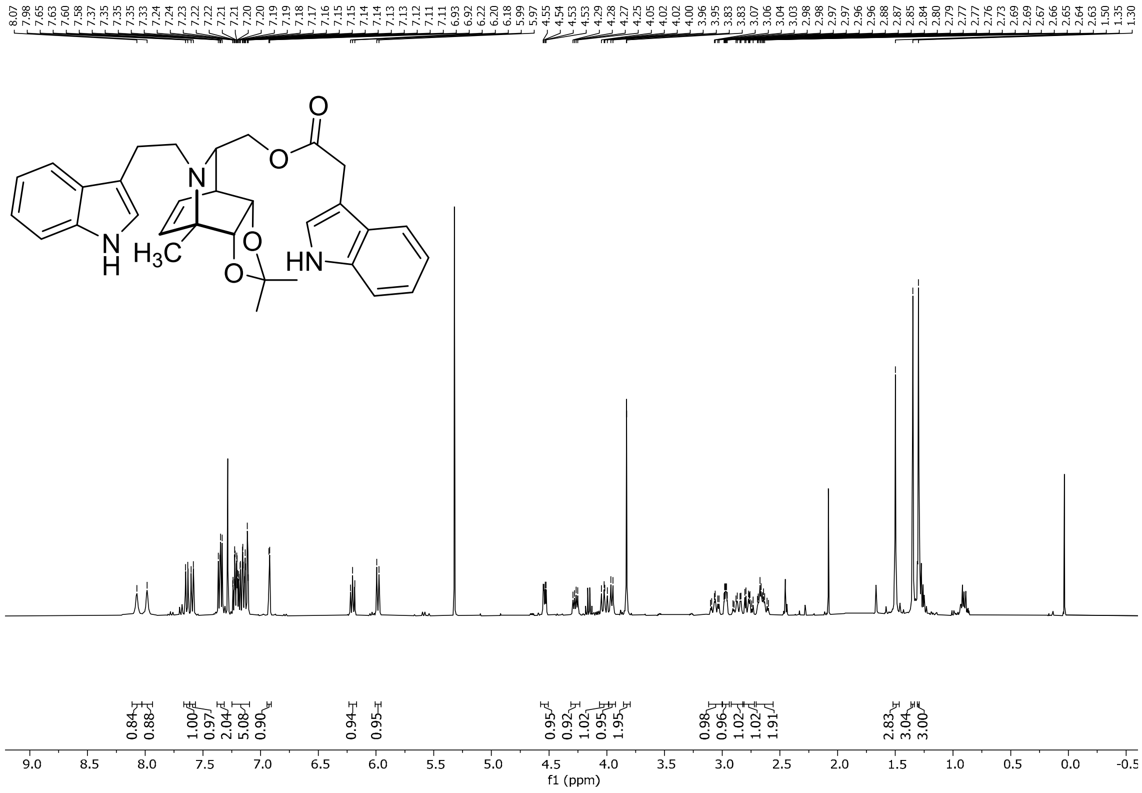

Figure S26. ^{1}H NMR of **15** in $CDCl_3$

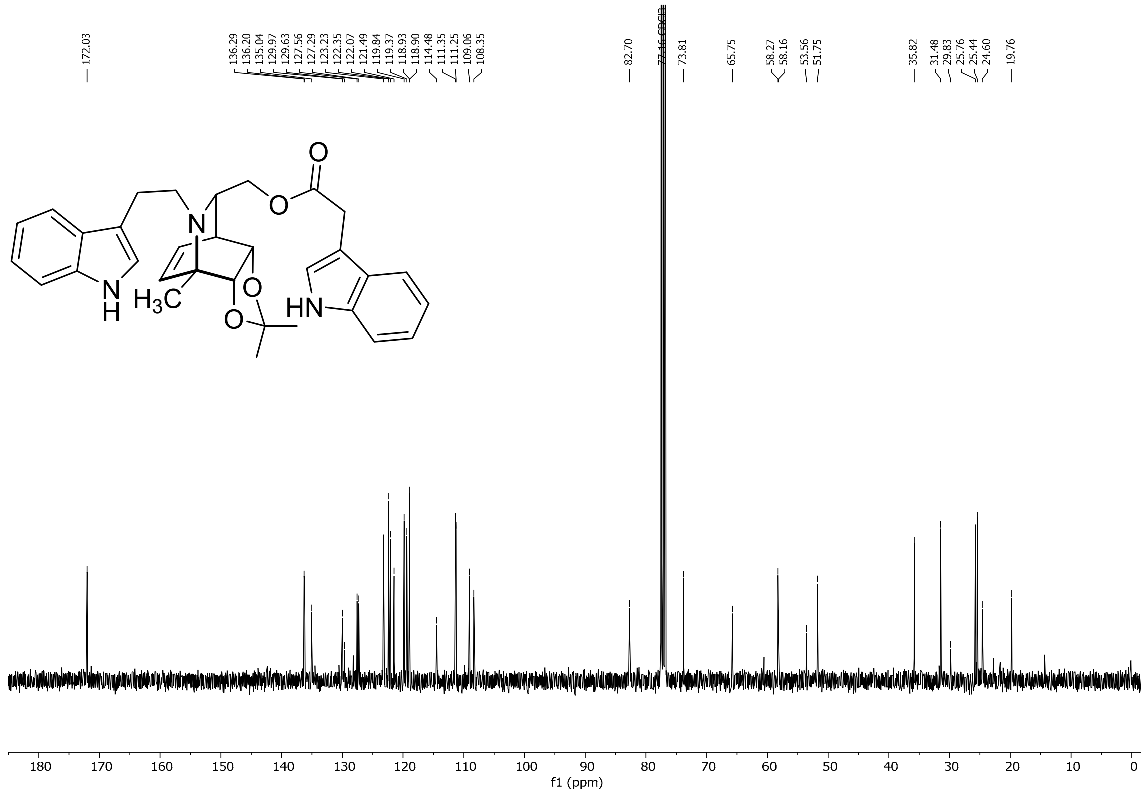

Figure S27. ^{13}C NMR of **15** in $CDCl_3$
